# Supplementary material for: USP9X regulates centrosome duplication and promotes breast carcinogenesis
Source: Nat Commun. 2017 Mar 31;8:14866. doi: 10.1038/ncomms14866 (PMC5380967; doi:10.1038/ncomms14866)
Supplement: Supplementary Information — Supplementary Figures and Supplementary Tables [file ncomms14866-s1.pdf]

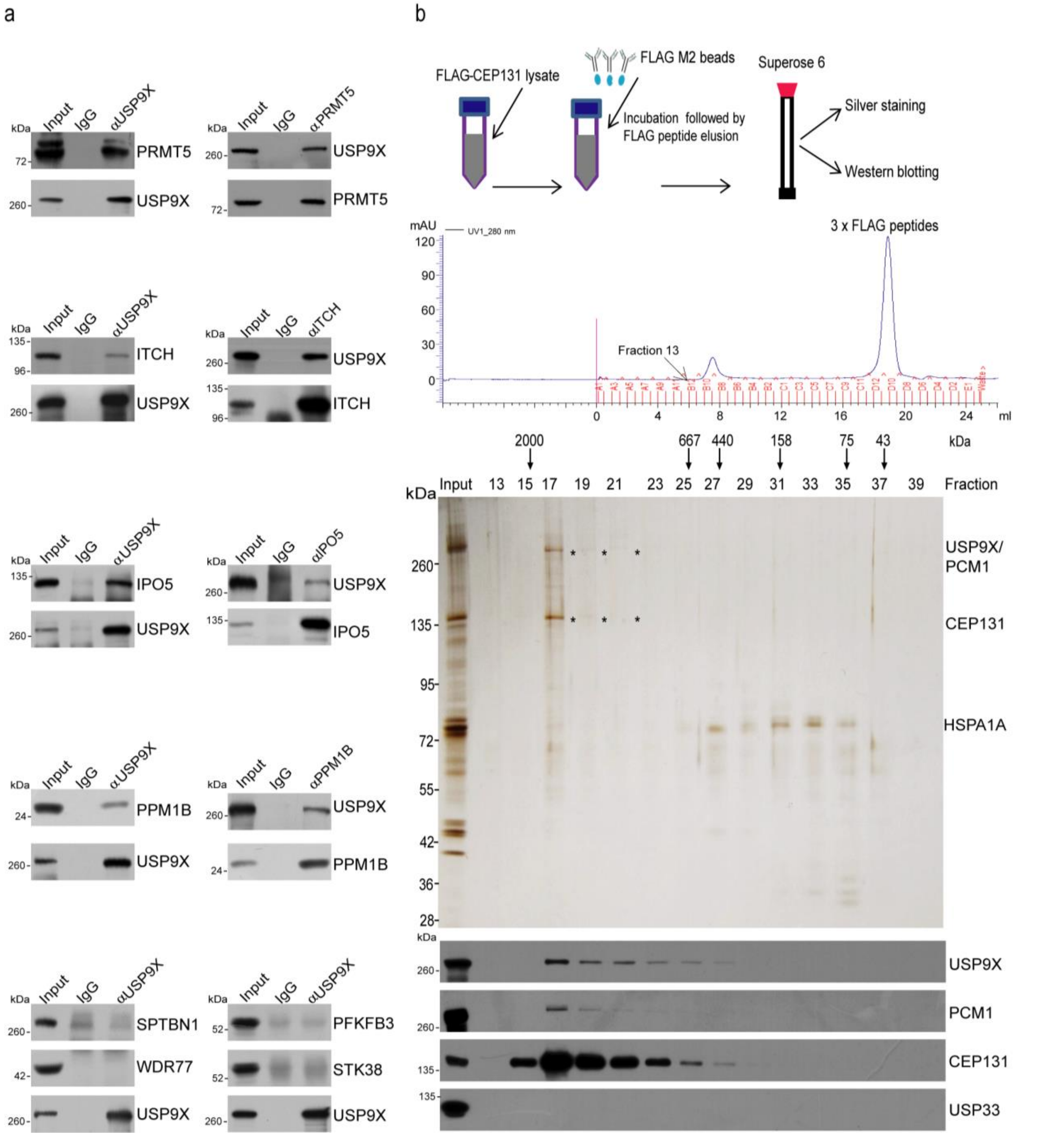

Supplementary Figure 1 (Figure 1 Continued). Deubiquitinase USP9X Is Physically Associated with the Centriolar Satellite Protein CEP131. (a) Whole cell lysates from HeLa cells were immunoprecipitated followed by immunoblotting with antibodies against the indicated proteins. (b) Silver staining and Western blotting analysis of the CEP131-containing complexes fractionated by Superose 6 gel filtration with antibodies against the indicated proteins. The elution positions of calibration proteins with known molecular masses are indicated, and an equal volume from each fraction was analyzed.

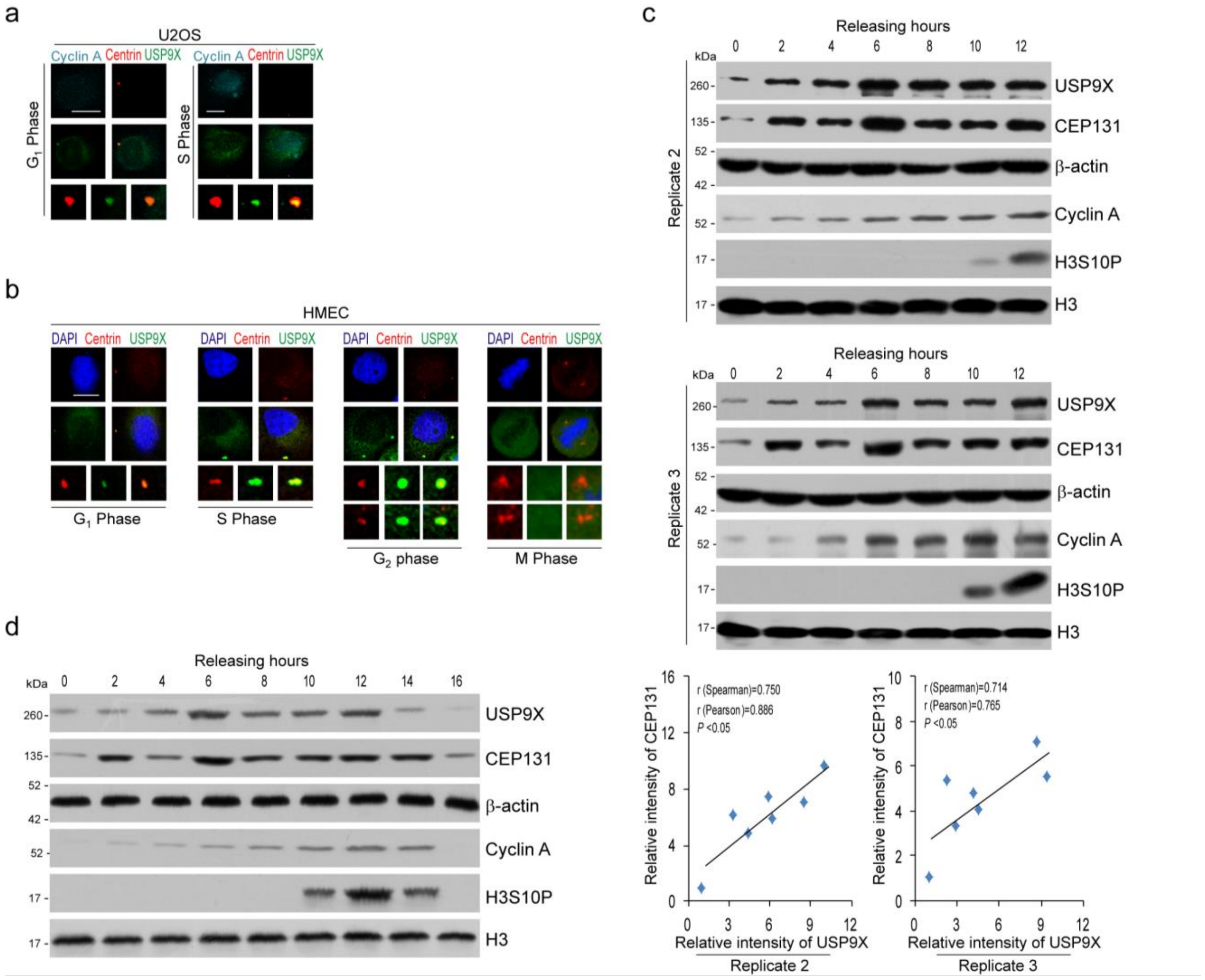

Supplementary Figure 2 (Figure 2 Continued). Co-localization of USP9X with CEP131 in Centrosome. (a) Confocal microscopy analysis of USP9X subcellular localization in G_1 and S phase. U2OS cells stably expressing CFP-cyclin A was fixed and stained with antibodies against Centrin and USP9X. Representative images from biological triplicate experiments are shown. Scale bar, 10 µm. (b) Human mammary epithelial cells (HMECs) were synchronized by double-thymidine block and then released to G_1-, S-, G_2-, or M phase followed by immunostaining with antibodies against USP9X and Centrin. Representative images from biological triplicate experiments are shown. Scale bar, 10 µm. (c) U2OS cells synchronized by double-thymidine block were released and cellular extracts were collected for Western blotting analysis with antibodies against the indicated proteins. Two sets of representative images from biological triplicate experiments are shown (Related to Fig. 2e). Intensity of each band was quantified by densitometry with Image J software with β-actin as a normalizer. The correlation coefficient and *P* values are shown. (d) U2OS cells synchronized by double-thymidine block were released and cellular extracts were collected at the indicated time for Western blotting analysis with antibodies against the indicated proteins.

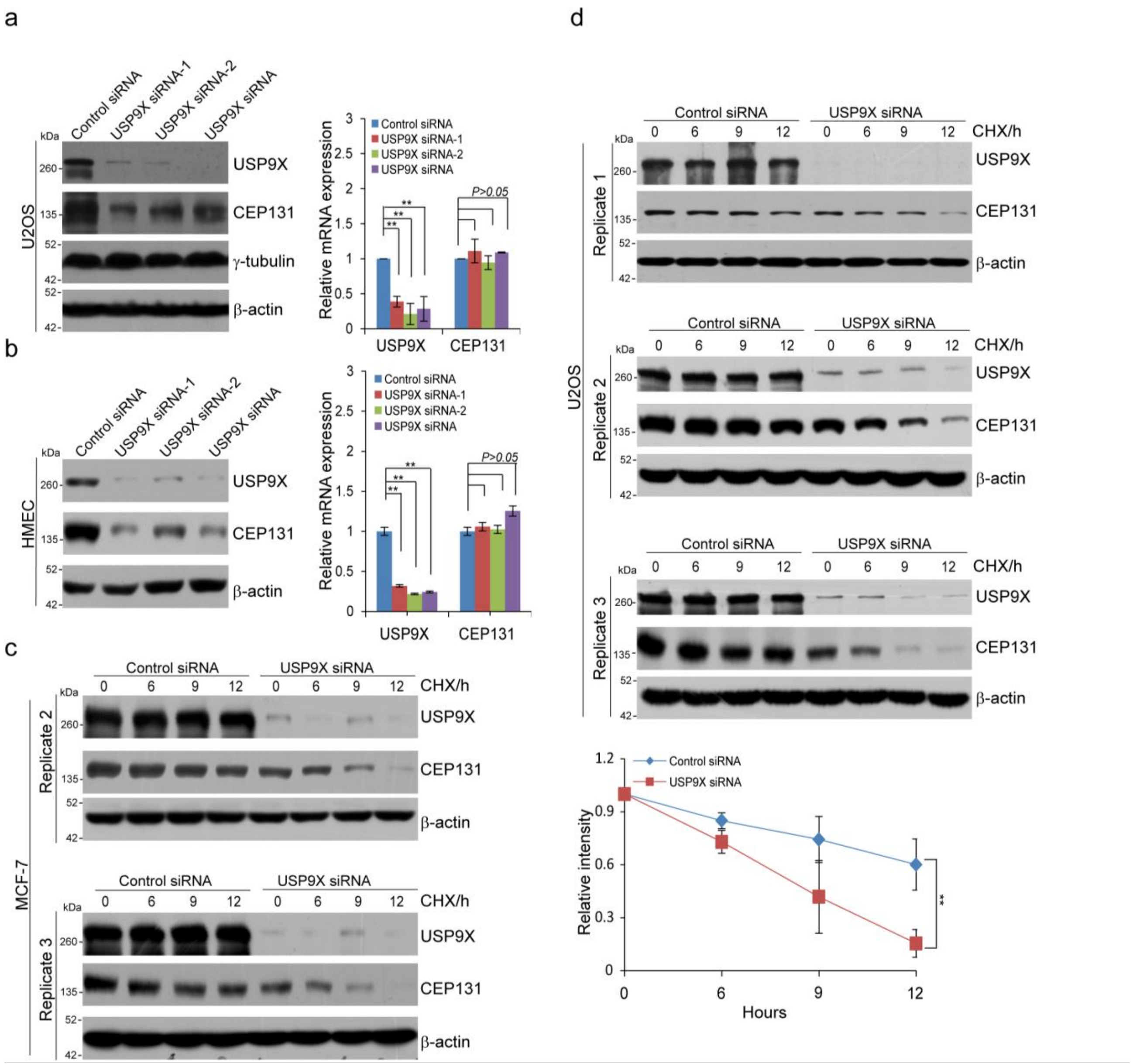

Supplementary Figure 3 (Figure 3 Continued). USP9X Promotes CEP131 Stabilization. (a) U2OS cells were transfected with control siRNA or different sets of USP9X siRNAs. Cellular extracts and total RNA were prepared and analyzed by Western blotting and qRT-PCR, respectively. Each bar represents the mean ± S.D. for biological triplicate experiments. *P* values were computed by one-way ANOVA in conjugation with Bonferroni's correction. $^{**}P<0.01$. (b) HMEC cells were transfected with control siRNA or different sets of USP9X siRNAs. Cellular extracts and total RNA were prepared and analyzed by Western blotting and qRT-PCR, respectively. Each bar represents the mean ± S.D. for biological triplicate experiments. *P* values were computed by one-way ANOVA in conjugation with Bonferroni's correction. $^{**}P<0.01$. (c) MCF-7 cells transfected with control siRNA or USP9X siRNA were treated with cycloheximide

(CHX) and harvested at the indicated time followed by Western blotting analysis. Two sets of representative images from biological triplicate experiments are shown (Related to Fig. 3d). (d) U2OS cells transfected with control siRNA or USP9X siRNA were treated with cycloheximide (CHX) and harvested at the indicated time followed by Western blotting analysis. Intensity of each band from biological triplicate experiments was quantified by densitometry with Image J software with β-actin as a normalizer. Each bar represents the mean ± S.D. for biological triplicate experiments. *P* values were computed by two-way ANOVA in conjugation with Bonferroni's correction. $^{**}P<0.01$.

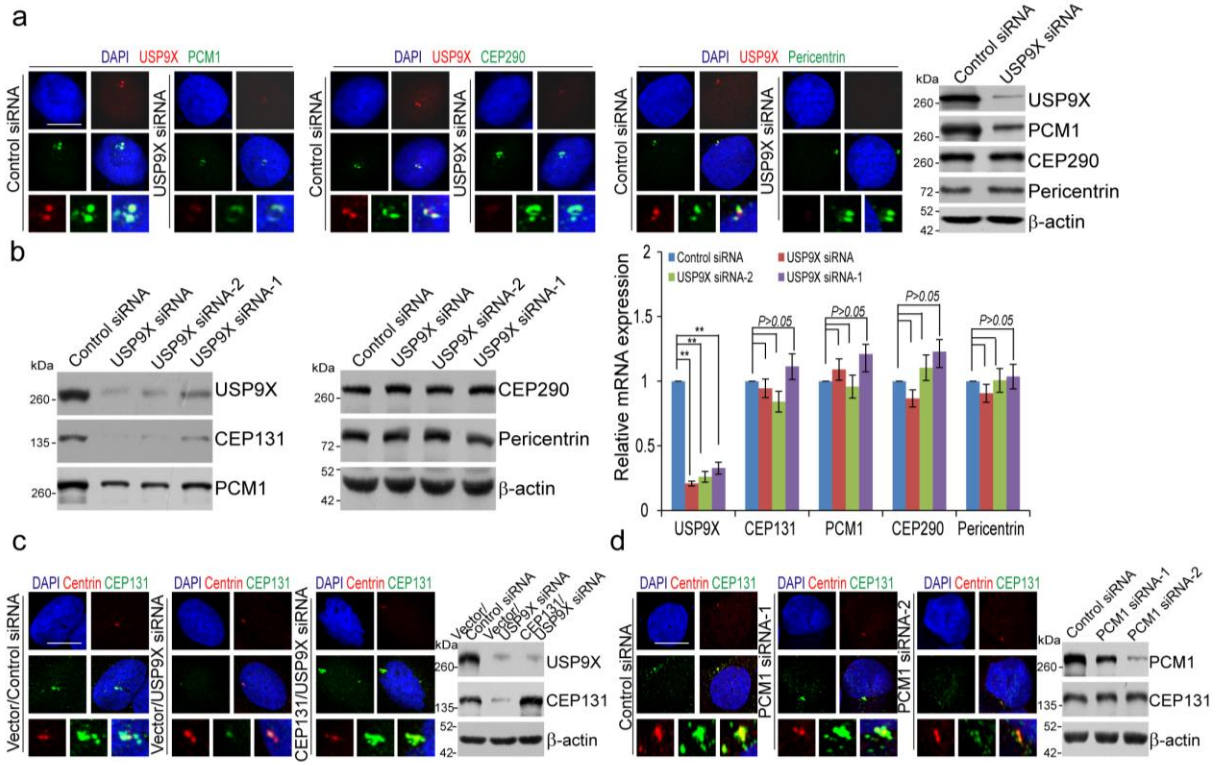

Supplementary Figure 4 (Figure 3 Continued). USP9X Depletion Was Associated with Decreased Protein Abundance and Centrosomal Localization of PCM1, but Not CEP290 or Pericentrin. (a) U2OS cells transfected with control siRNA or USP9X siRNA were fixed and subjected to immunostaining with antibodies against the indicated proteins. Representative images from biological triplicate experiments are shown. Scale bar, 10 μm. Cellular extracts were prepared and analyzed by Western blotting. (b) U2OS cells were transfected with control siRNA or different sets of USP9X siRNAs. Cellular extracts were prepared and analyzed by Western blotting and qRT-PCR. Each bar represents the mean ± S.D. for biological triplicate experiments. *P* values were computed by one-way ANOVA in conjugation with Bonferroni's correction. $^{**}P<0.01$. (c) USP9X depleted cells were transfected with control vector or FLAG-CEP131, and cells were fixed and immunostained with antibodies against the indicated proteins. Representative images from biological triplicate experiments are shown. Scale bar, 10 μm. The expression of indicated proteins was examined by Western blotting. (d) U2OS cells transfected with control siRNA or PCM1 siRNAs were fixed and subjected to immunostaining with antibodies against the indicated proteins. Representative images from biological triplicate experiments are shown. Scale bar, 10 μm. Cellular extracts were prepared and analyzed by Western blotting.

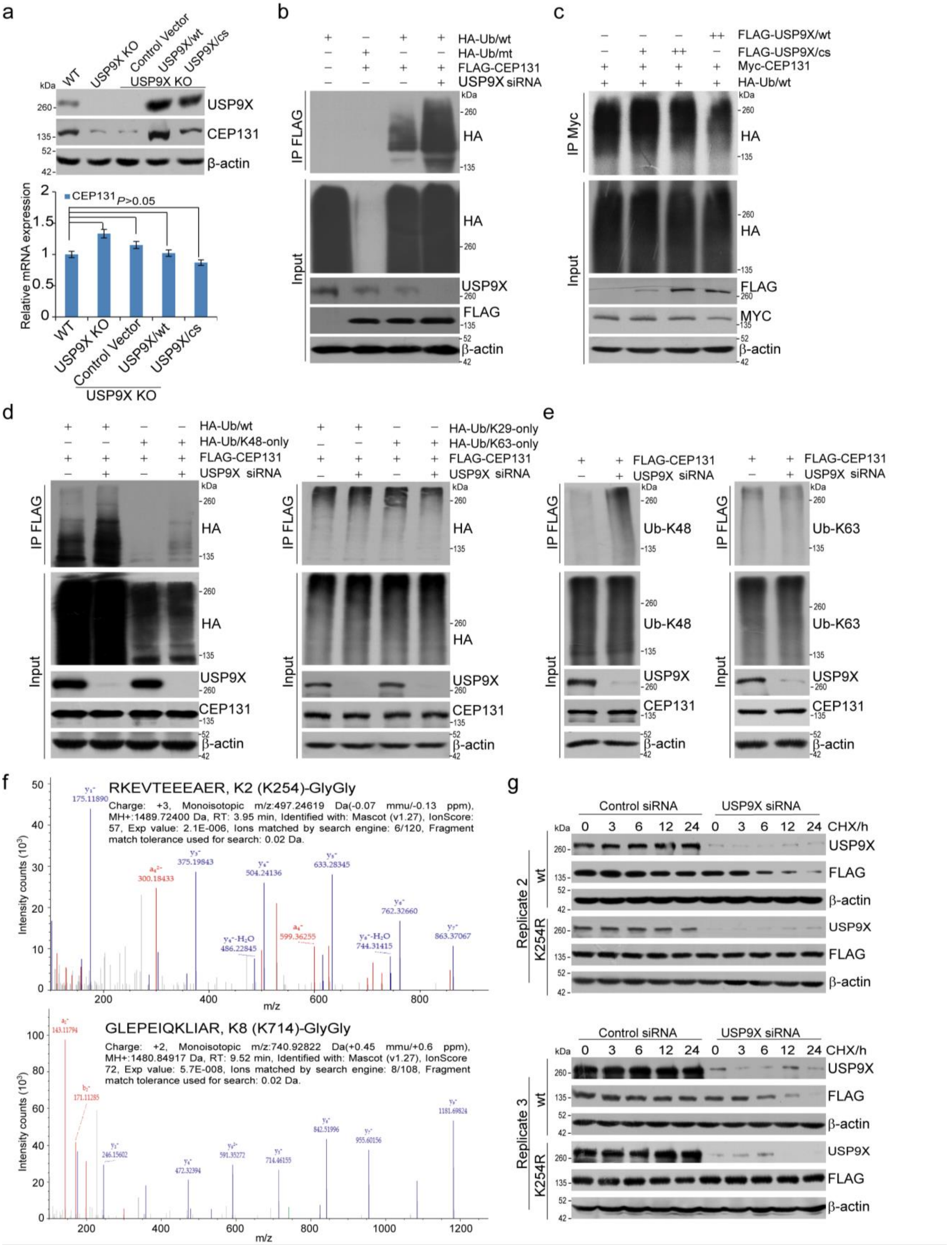
a
WT
USP9X KO
Control Vector
USP9X/wt
USP9X/cs
USP9X KO
kDa
260
135
52
42
USP9X
CEP131
β-actin
CEP131
P>0.05
Relative mRNA expression
2
1.5
1
0.5
0
WT
USP9X KO
Control Vector
USP9X/wt
USP9X/cs
USP9X KO
b
HA-Ub/wt
HA-Ub/mt
FLAG-CEP131
USP9X siRNA
IP FLAG
Input
HA
USP9X
FLAG
β-actin
c
FLAG-USP9X/wt
FLAG-USP9X/cs
Myc-CEP131
HA-Ub/wt
IP Myc
Input
HA
FLAG
MYC
β-actin
d
HA-Ub/wt
HA-Ub/K48-only
FLAG-CEP131
USP9X siRNA
HA-Ub/K29-only
HA-Ub/K63-only
IP FLAG
Input
HA
USP9X
CEP131
β-actin
e
FLAG-CEP131
USP9X siRNA
IP FLAG
Input
Ub-K48
Ub-K63
USP9X
CEP131
β-actin
f
RKEVTEEEAER, K2 (K254)-GlyGly
Charge: +3, Monoisotopic m/z:497.24619 Da(-0.07 mmu/-0.13 ppm), MH+:1489.72400 Da, RT: 3.95 min, Identified with: Mascot (v1.27), IonScore: 57, Exp value: 2.1E-006, Ions matched by search engine: 6/120, Fragment match tolerance used for search: 0.02 Da.
Intensity counts (10³)
m/z
GLEPEIQKLIAR, K8 (K714)-GlyGly
Charge: +2, Monoisotopic m/z:740.92822 Da(+0.45 mmu/+0.6 ppm), MH+:1480.84917 Da, RT: 9.52 min, Identified with: Mascot (v1.27), IonScore 72, Exp value: 5.7E-008, Ions matched by search engine: 8/108, Fragment match tolerance used for search: 0.02 Da.
Intensity counts (10³)
m/z
g
Control siRNA
USP9X siRNA
CHX/h
0 3 6 12 24 0 3 6 12 24
Replicate 2
Replicate 3
wt
K254R
USP9X
FLAG
β-actin

Supplementary Figure 5 (Figure 4 Continued). USP9X Promotes CEP131 Deubiquitination. (a) Wild type and USP9X knockout HMEC cells were transfected with control vector, FLAG-USP9X/wt or FLAG-USP9X/C1566S. Cellular extracts were prepared and analyzed by Western blotting and qRT-PCR. Each bar represents the mean $\pm$ S.D. for biological triplicate experiments. *P* values were computed by one-way ANOVA in conjugation with Bonferroni's correction. (b) HeLa cells stably expressing FLAG-CEP131 were co-transfected with control siRNA or USP9X siRNA together with HA-Ub/wt or HA-Ub/mt as indicated. Cellular extracts were prepared for co-immunoprecipitation assays with anti-FLAG followed by IB with anti-HA. (c) HeLa cells with Dox-inducible expression of USP9X/wt or USP9X/C1566S cultured in the absence or presence of Dox were co-transfected with Myc-CEP131 and HA-Ub/wt. Cellular extracts were prepared for co-immunoprecipitation assays with anti-Myc followed by IB with anti-HA. (d) HeLa cells stably expressing FLAG-CEP131 were co-transfected with control siRNA or USP9X siRNA together with HA-Ub/K29-only, HA-Ub/K48-only or HA-Ub/K63-only as indicated. Cellular extracts were prepared for co-immunoprecipitation assays with anti-FLAG followed by IB with anti-HA. (e) HeLa cells stably expressing FLAG-CEP131 were transfected with control siRNA or USP9X siRNA. Cellular extracts were prepared for co-immunoprecipitation assays with antibodies as indicated. (f) Mass spectrometry analysis of CEP131 ubiquitin conjugation sites. HeLa cells stably expressing FLAG-CEP131 were transfected with HA-Ub and cellular extracts were collected and sequentially purified with anti-FLAG affinity gel and HA affinity gel to enrich HA-Ub conjugated CEP131. The retrieved protein was subjected to mass spectrometry analysis. Fragmentation spectrums and parameters of the identified CEP131-Ub peptides are shown. (g) MCF-7 cells stably expressing CEP131/wt or CEP131/K254R mutant were transfected with control siRNA or USP9X siRNA. Cells were treated with CHX and harvested at the indicated time followed by Western blotting analysis. Two sets of representative images from biological triplicate experiments are shown (Related to Fig. 4g).

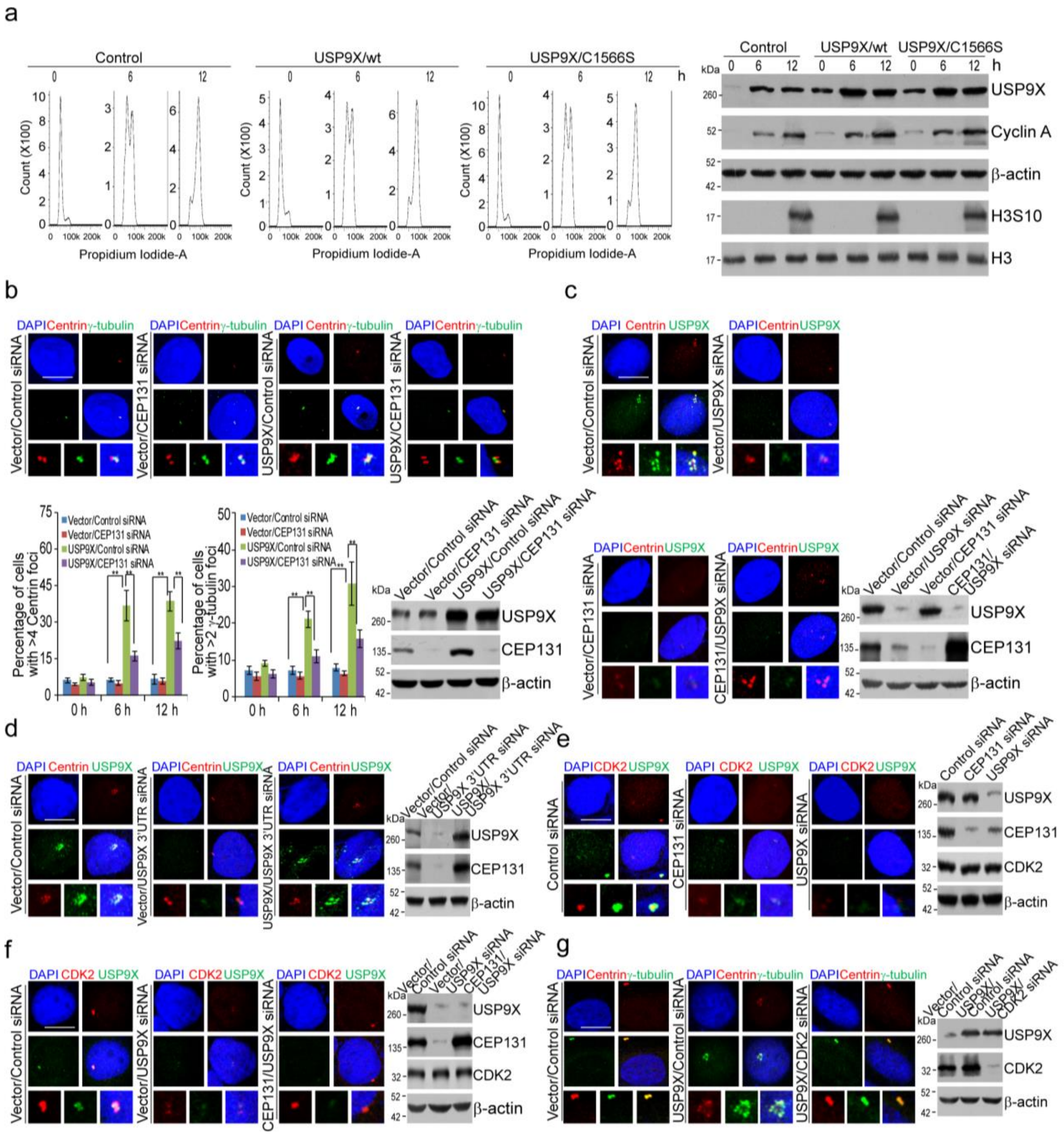

Supplementary Figure 6 (Figure 5 Continued). USP9X/CEP131 Regulates Centrosome Amplification and Chromosome Stability. (a) Control U2OS cells and U2OS cells with Dox-inducible expression of USP9X/wt or USP9X/C1566S in the presence of Dox were synchronized with double thymidine block and released. Cell cycle profiles of these cells were determined by FACS and Western blotting. (b) MCF-7 cells stably expressing USP9X were transfected with

control siRNA or CEP131 siRNA as indicated. The cells were synchronized with double thymidine block and released followed by immunostaining with antibodies against the indicated proteins (upper panel). Scale bar, 10 μm. Population of cells with the indicated numbers of foci at different time points were counted (lower panel). Each bar represents the mean ± S.D. for biological triplicate experiments **P<0.01, one-way ANOVA. (c) HMEC cells transfected with siRNAs and/or expressing vectors as indicated were treated with hydroxyurea (HU) followed by analysis of centrosome numbers with immunostaining. Representative images from biological triplicate experiments are shown. Scale bar, 10 μm. (d) Control U2OS cells or U2OS cells stably expressing USP9X were transfected with control siRNA or *USP9X* 3'UTR siRNA for 96 hours followed by HU treatment and immunostaining with antibodies against the indicated proteins. Representative images from biological triplicate experiments are shown. Scale bar, 10 μm. (e) U2OS cells transfected with control siRNA, USP9X or CEP131 siRNA were fixed and subjected to immunostaining with antibodies against the indicated proteins. Representative images from biological triplicate experiments are shown. Scale bar, 10 μm. (f) USP9X-depleted U2OS cells transfected with control vector or FLAG tagged CEP131 were fixed and subjected to immunostaining with antibodies against the indicated proteins. Representative images from biological triplicate experiments are shown. Scale bar, 10 μm. (g) U2OS cells with Dox-inducible expression of FLAG-USP9X were transfected with control siRNA or CDK2 siRNA, synchronized with double thymidine block and released. The cells were collected 6 hours post releasing, fixed and subjected to immunostaining with antibodies against the indicated proteins. Representative images from biological triplicate experiments are shown. Scale bar, 10 μm. The expression of indicated proteins was examined by Western blotting in (b), (c), (d), (e), (f) and (g) .

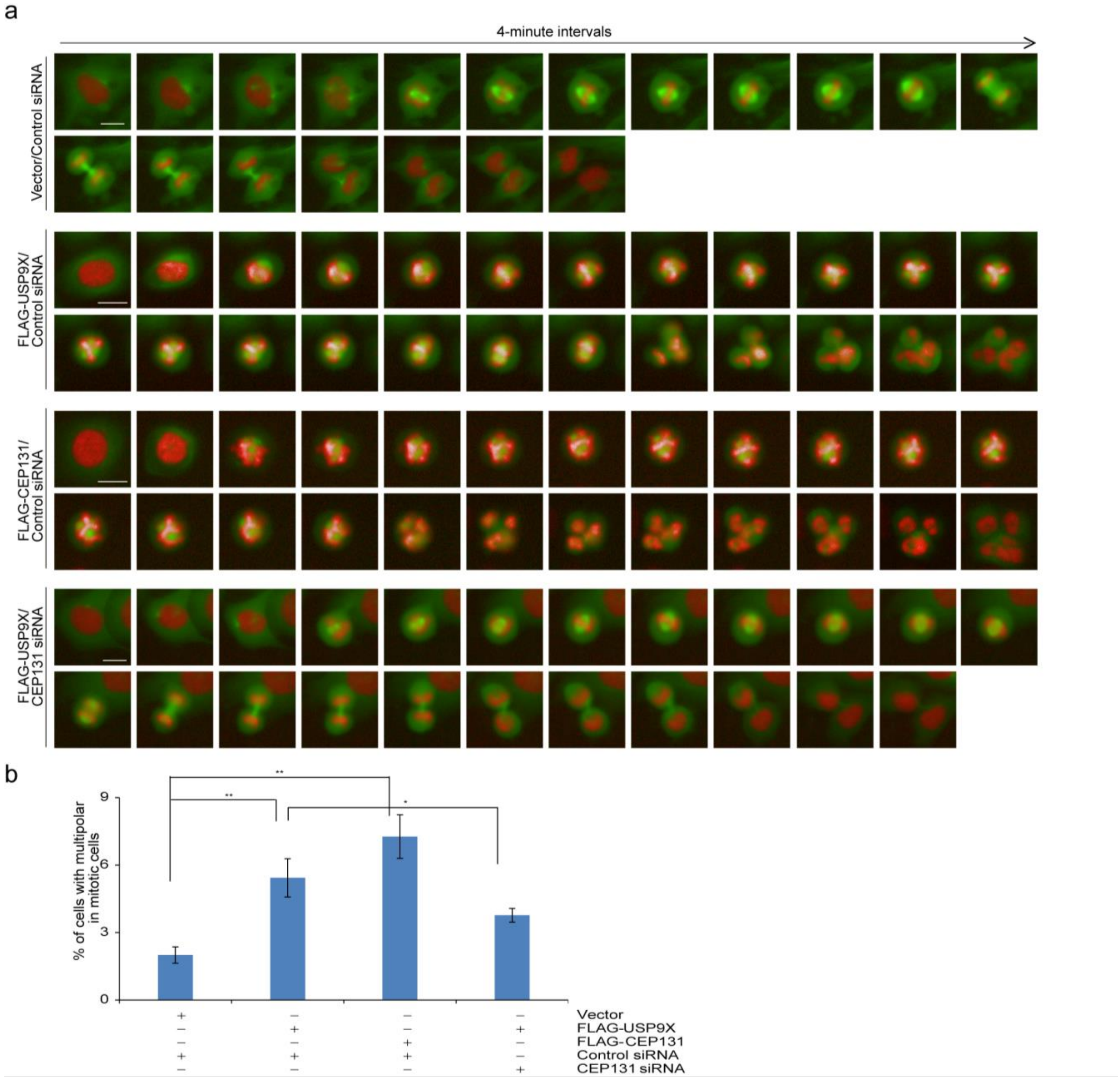

Supplementary Figure 7 (Figure 5 Continued). Live Cell Imaging Analysis of Mitotic Aberrations Associated with USP9X/CEP131 Alterations. (a) U2OS cells stably expressing dsRed-H2A and GFP-a-tubulin were transfected with indicated genes or siRNAs followed by high content live cell imaging analysis. Representative images from biological triplicate experiments under 40 × magnification fields (objective lens) with 4-minute intervals are shown. Scale bar, 10 μm. (b) Percentage of cells with multipolar mitotic phenotype was counted in (a). Each bar represents the mean ± S.D. for biological triplicate experiments. $^{*}P<0.05$, $^{**}P<0.01$, one-way ANOVA.

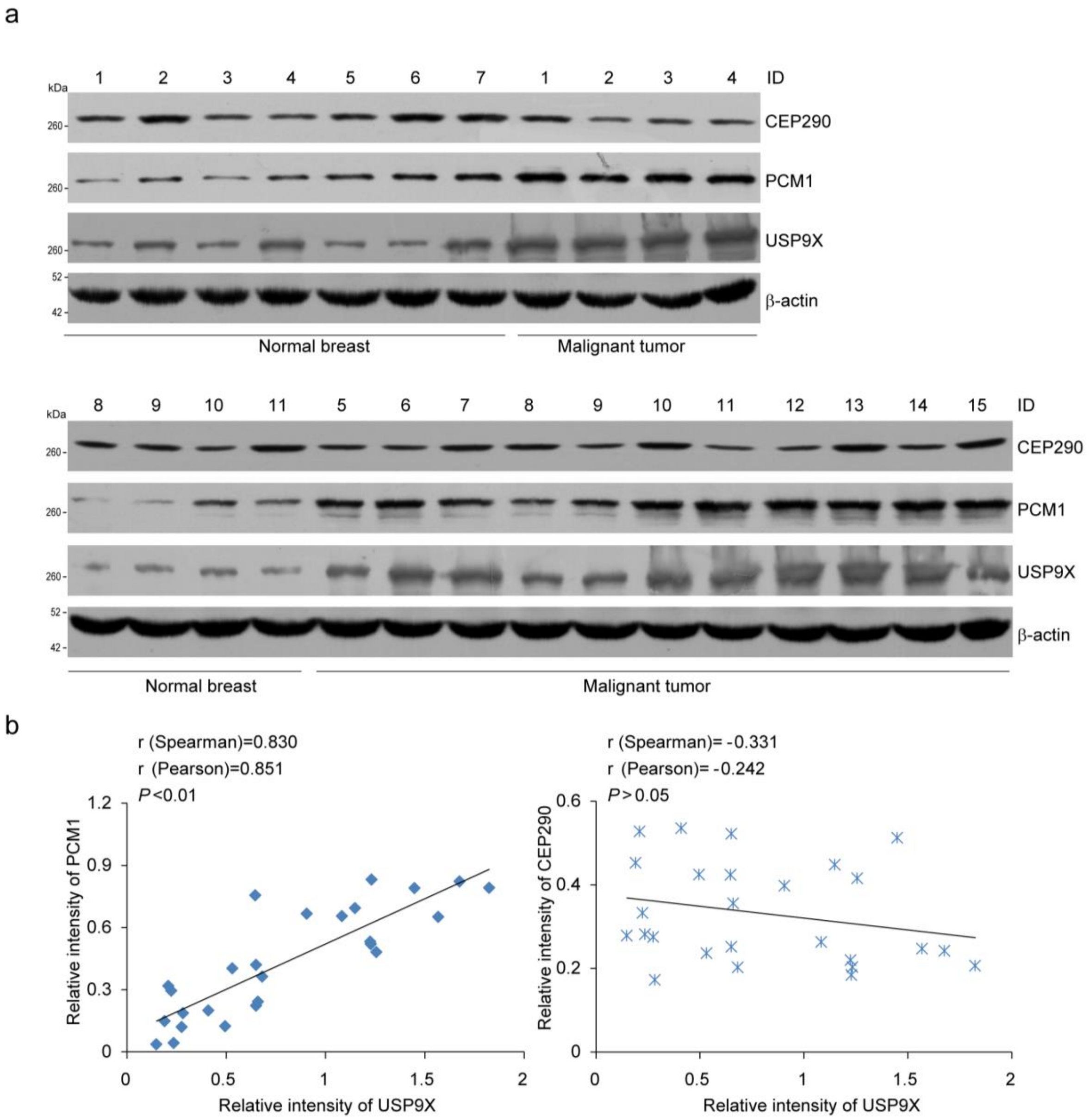

Supplementary Figure 8 (Figure 6 Continued). The Expression of PCM1 Is Elevated and Correlated with USP9X in Breast Cancer. (a) Cellular extracts from 11 normal mammary tissues and 15 breast cancer samples were prepared for Western blotting analysis with antibodies against the indicated proteins. (b) Intensity of each band in (a) was quantified by densitometry with Image J software with β-actin as a normalizer. The correlation coefficient and *P* values are shown.

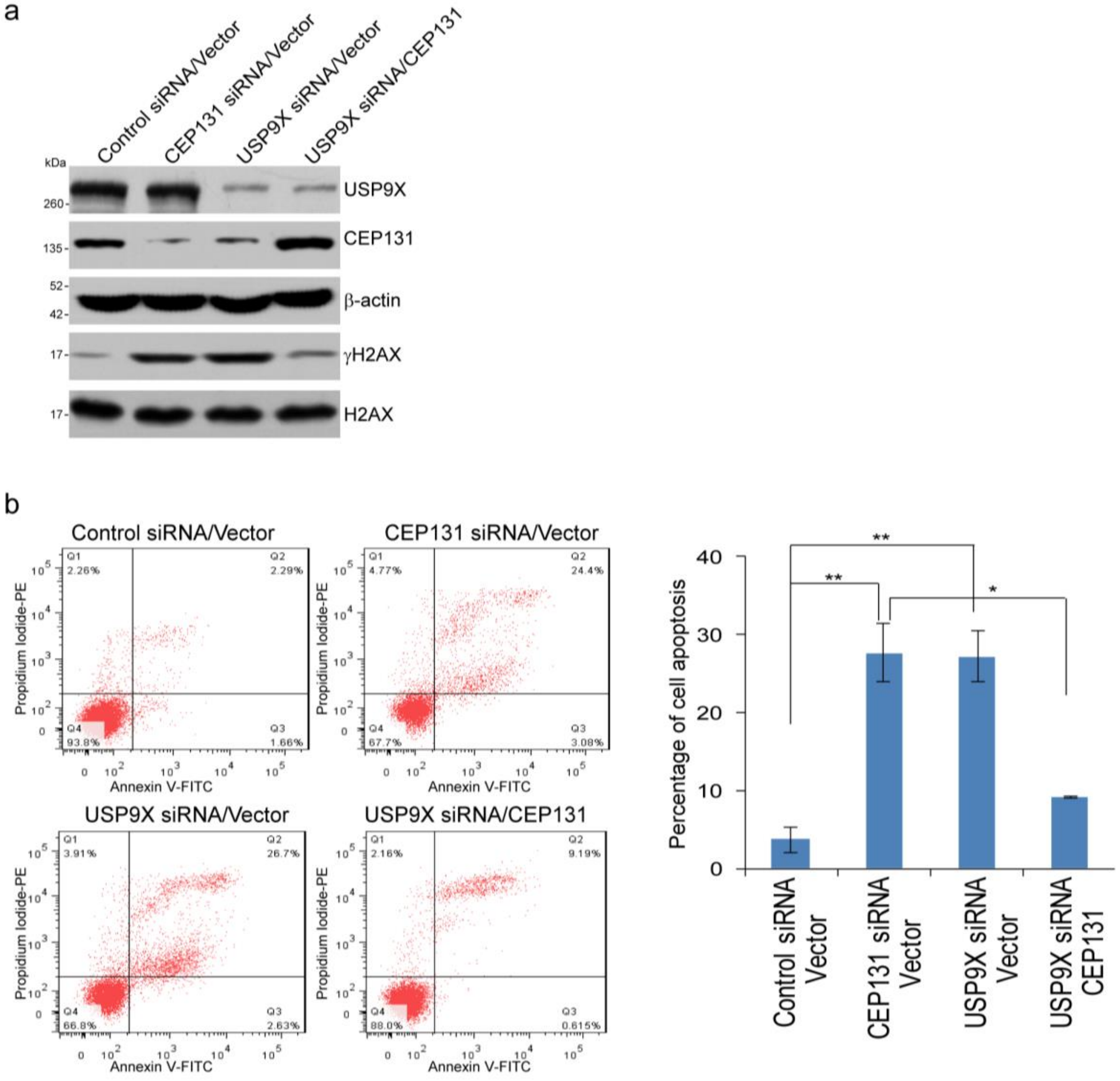

Supplementary Figure 9 (Figure 7 Continued). USP9X Promotes Breast Carcinogenesis through Regulating CEP131 Abundance. (a) MCF-7 cells transfected with indicated siRNAs and vectors encoding genes were collected for Western blotting analysis with antibodies against the indicated proteins. (b) MCF-7 cells transfected with indicated siRNAs and control vector or FLAG tagged CEP131 were stained with PI and Annexin V followed by FACS analysis. Representative images are shown. Each bar represents the mean ± S.D. for biological triplicate experiments. *P* values were computed by one-way ANOVA in conjugation with Bonferroni's correction. $^{*}P$<0.05; $^{**}P$<0.01.

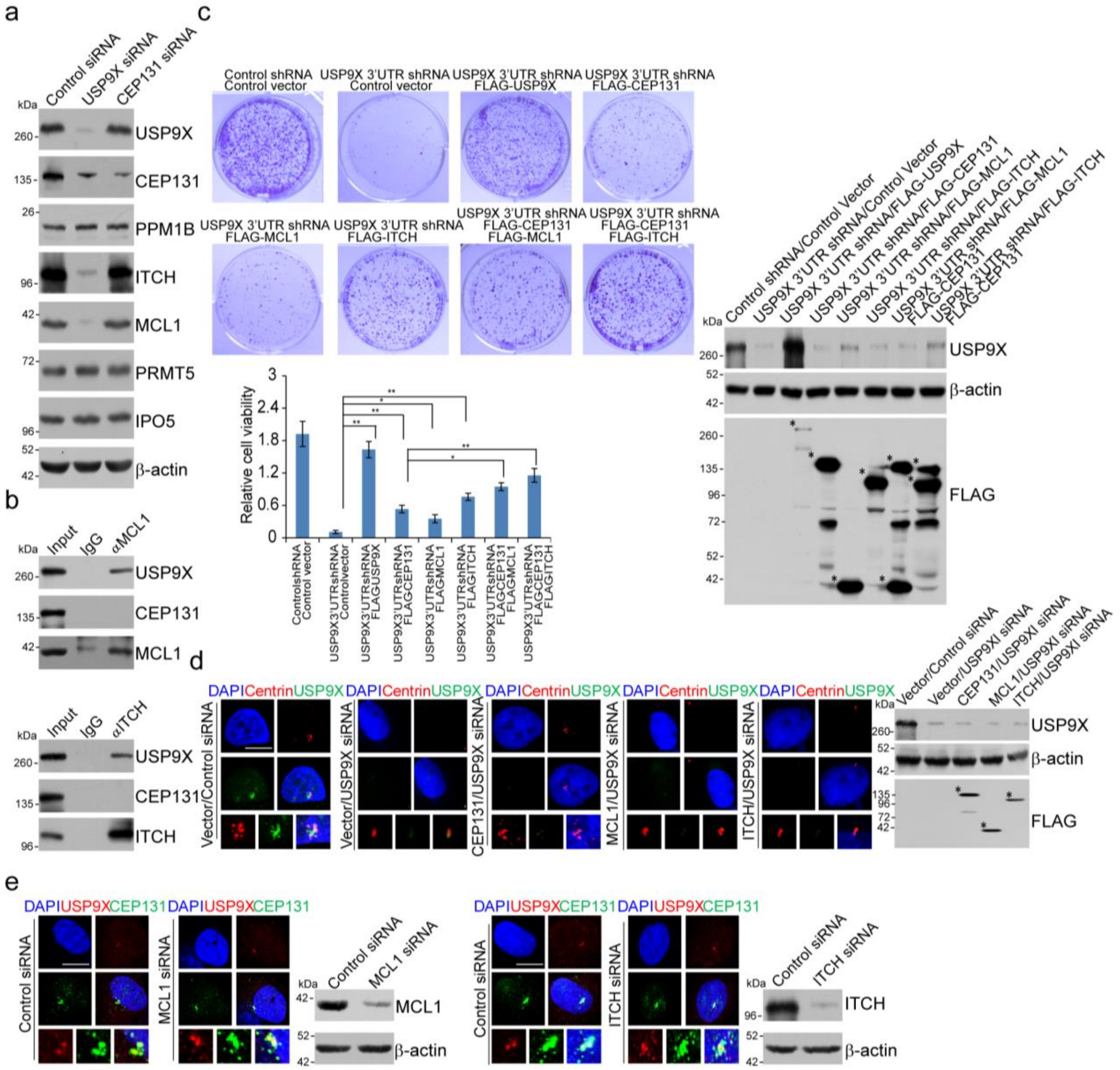

Supplementary Figure 10 (Figure 7 Continued). USP9X-Promoted CEP131 Stabilization Functions Independent of ITCH and MCL1 in Breast Cancer Cell Survival and Centrosome Biogenesis. (a) Cellular extracts from MCF-7 cells transfected with control siRNA, USP9X siRNA or CEP131 siRNA were prepared and analyzed by Western blotting with antibodies against the indicated proteins. (b) Whole cell lysates from MCF-7 cells were immunoprecipitated followed by immunoblotting with antibodies against the indicated proteins. (c) Colony formation assays with MCF-7 cells stably expressing shRNAs and FLAG tagged genes as indicated. Representative images are shown (upper panel). Each bar represents the mean ± S.D. for biological triplicate experiments (lower panel). $^{*}P<0.05$; $^{**}P<0.01$, one-way ANOVA. The expression of indicated proteins was examined by Western blotting. The asterisk indicates overexpressed proteins. (d) U2OS cells co-transfected with control siRNA or USP9X siRNA and FLAG tagged CEP131, ITCH or MCL1 were treated with hydroxyurea (HU)

followed by immunostaining with antibodies against the indicated proteins. Representative images from biological triplicate experiments are shown. Scale bar, 10 μm. The expression of indicated proteins was examined by Western blotting. The asterisk indicates overexpressed proteins. (e). U2OS cells transfected with control siRNA, ITCH siRNA or MCL1 siRNA were immunostained with antibodies against the indicated proteins. Representative images from biological triplicate experiments are shown. Scale bar, 10 μm. The expression of indicated proteins was examined by Western blotting.

Uncropped blots related to Figure 1b

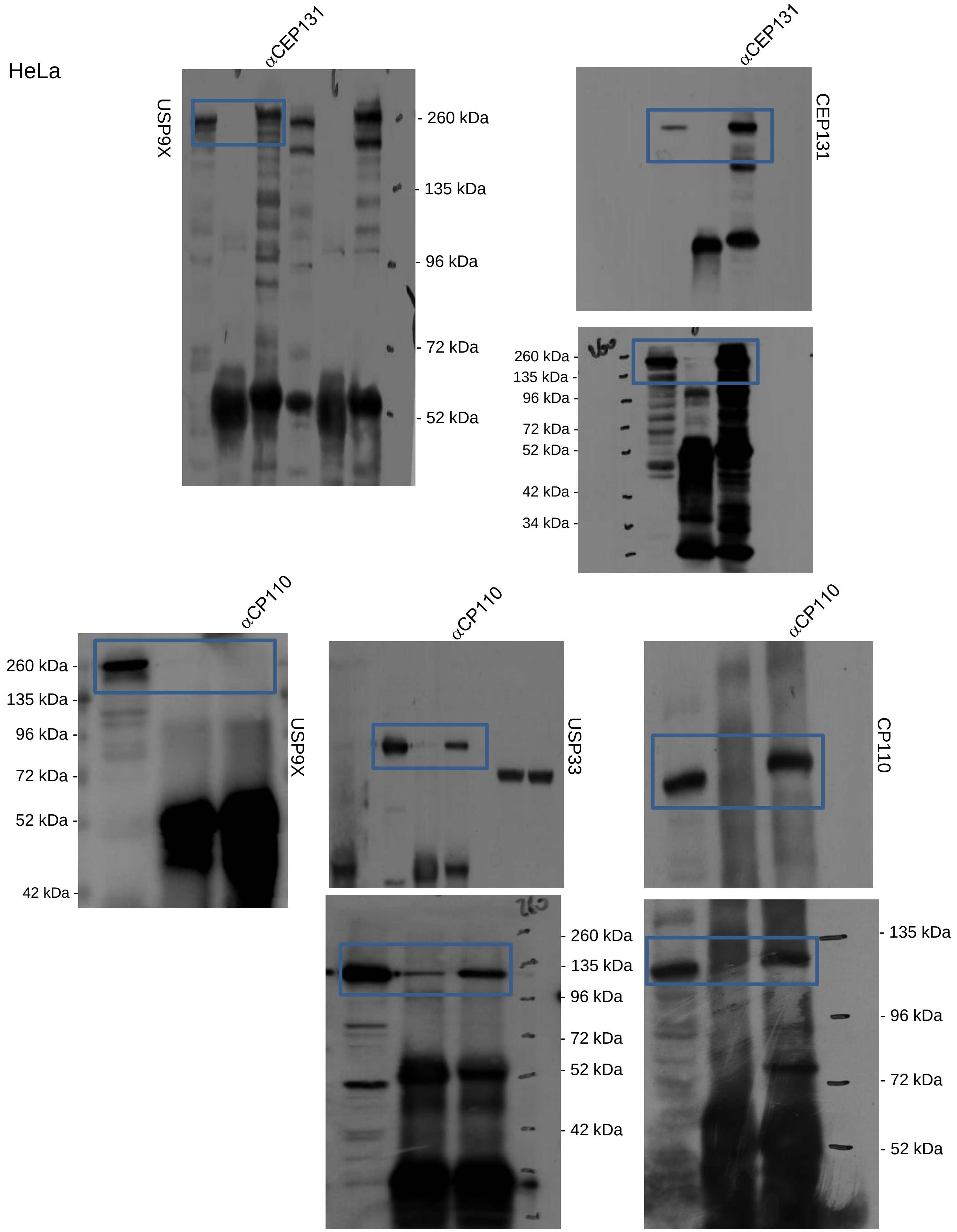

Uncropped blots related to Figure 1b

HeLa

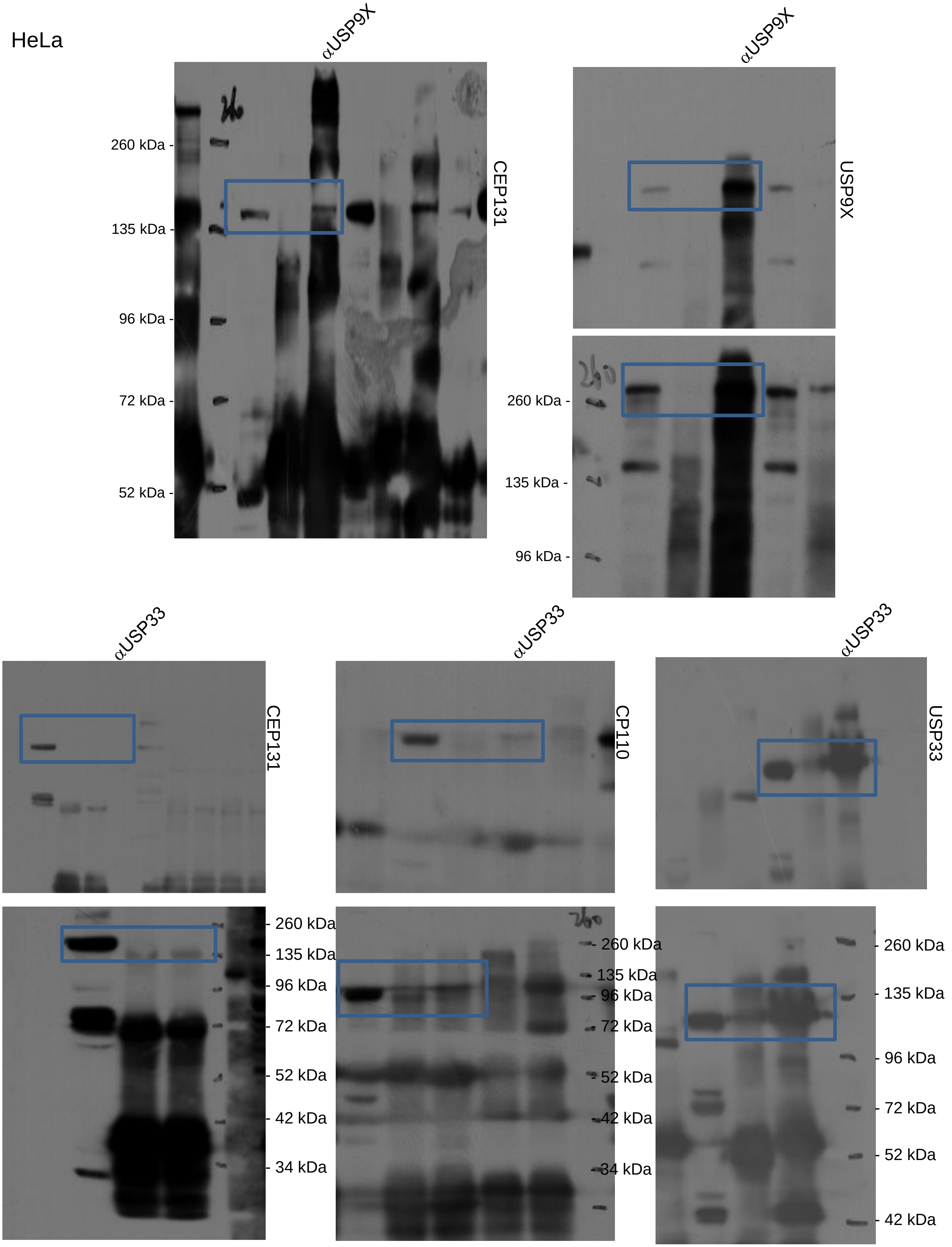

Uncropped blots related to Figure 1b

MCF-7

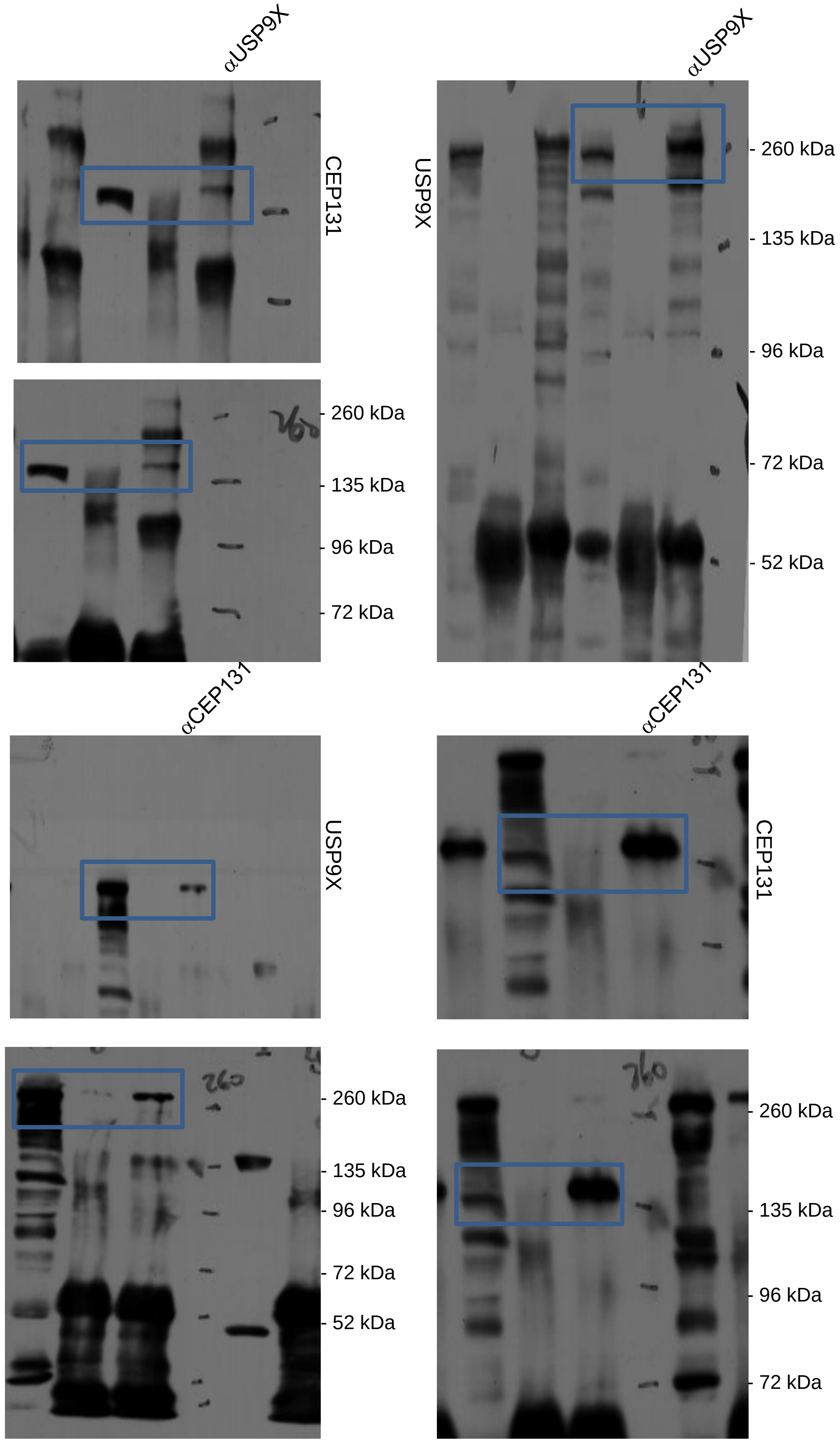

Uncropped blots related to Figure 1b

HEK 293T

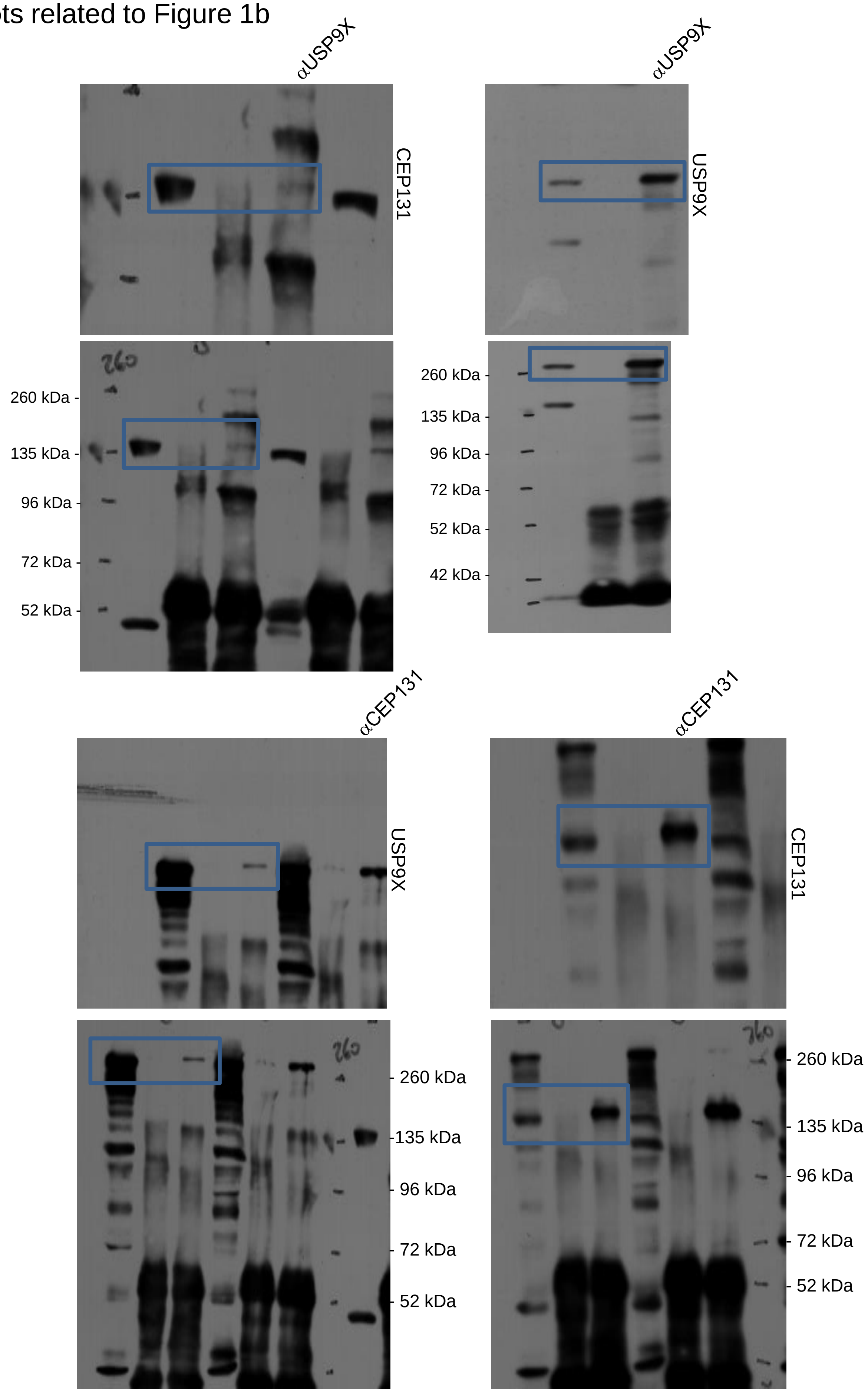

Uncropped blots related to Figure 1b

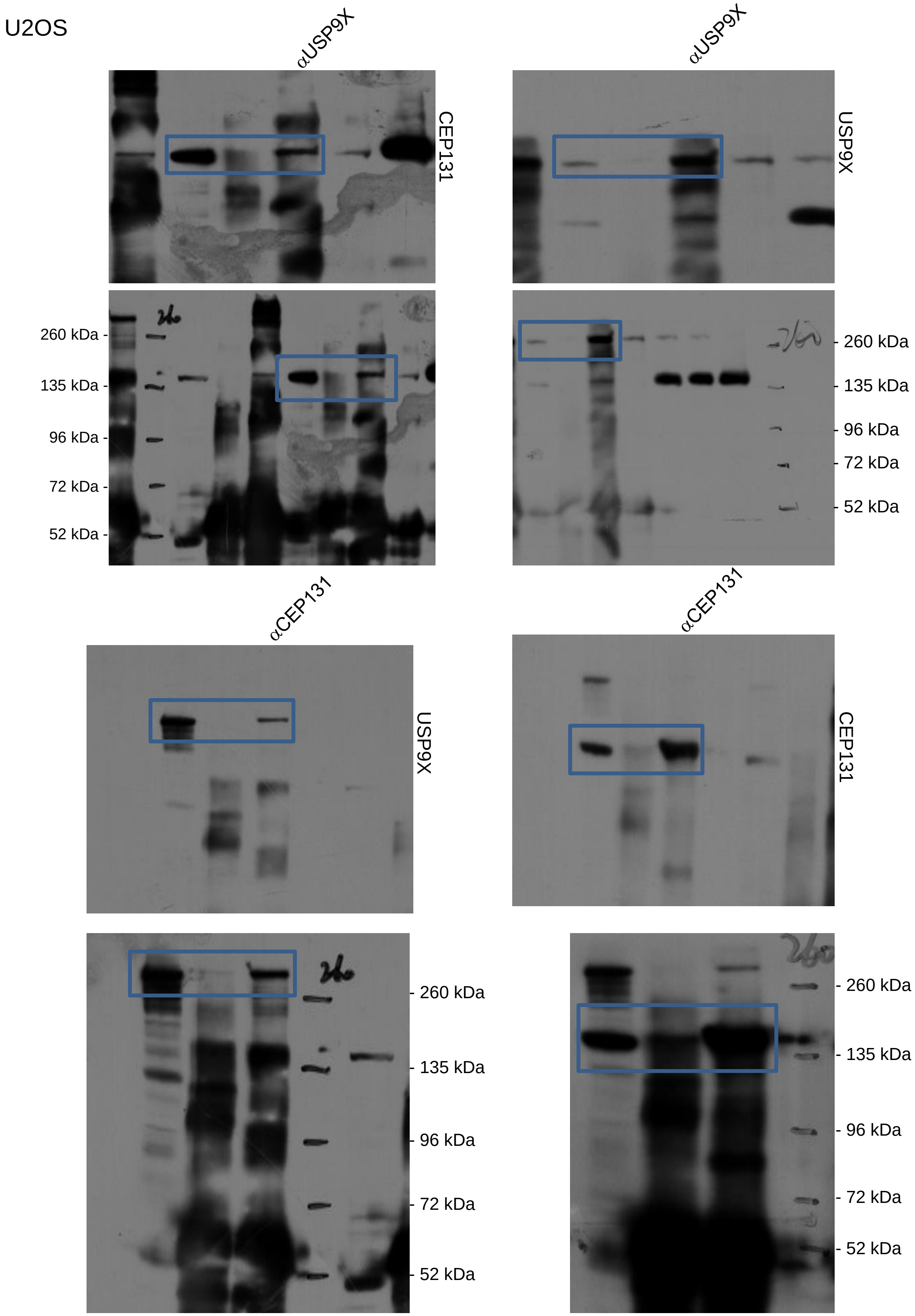

Uncropped blots related to Figure 1d

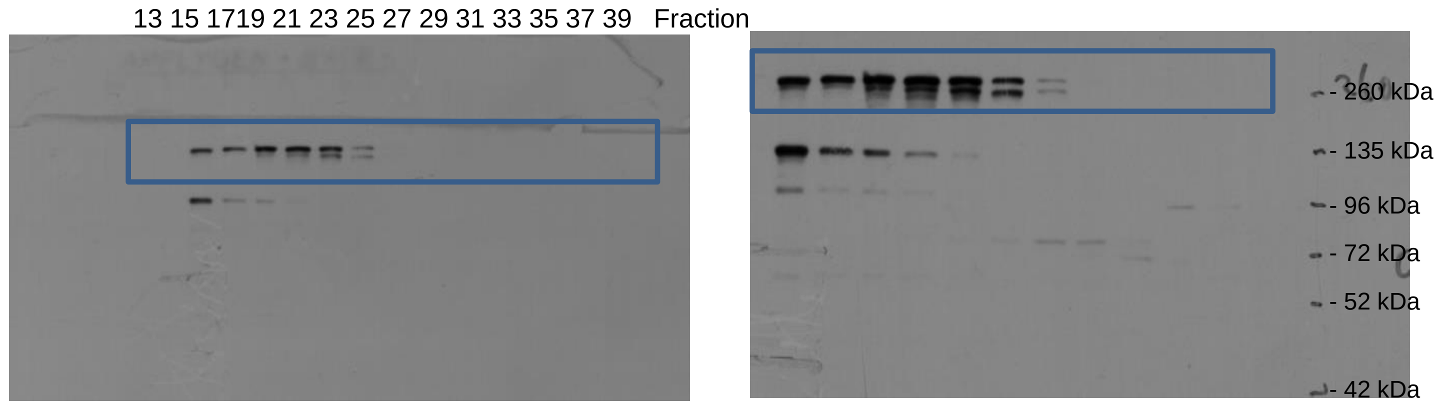

USP9X

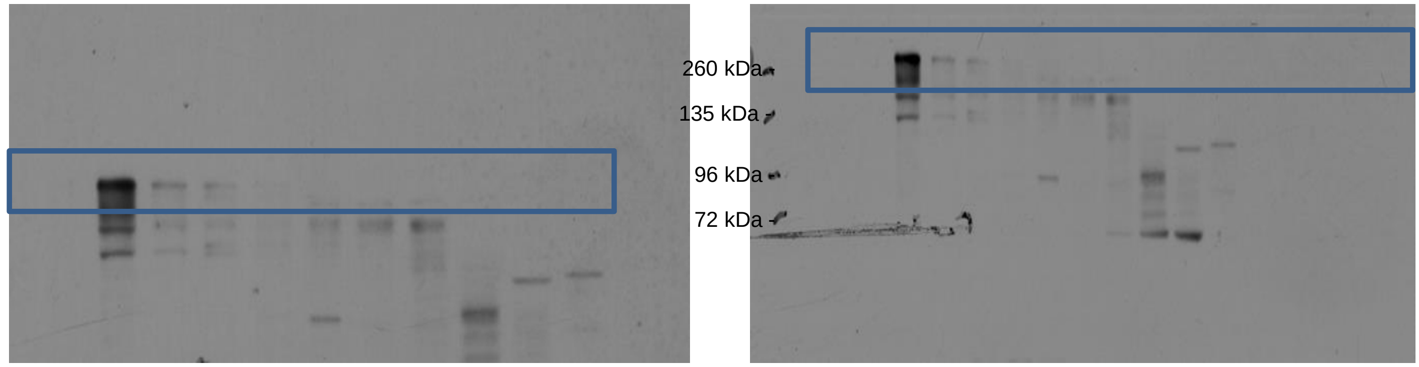

PCM1

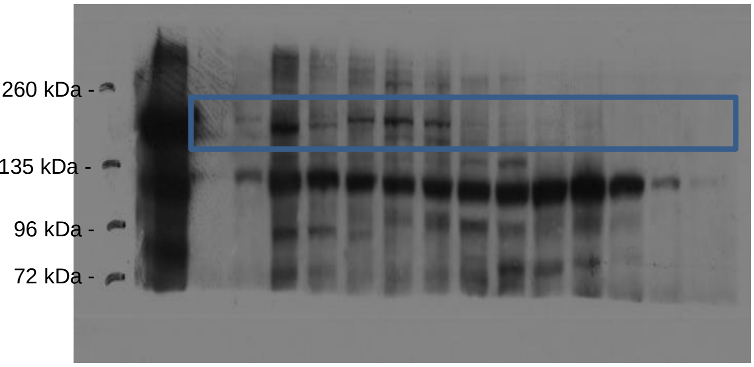

CEP131

Uncropped blots related to Figure 1e

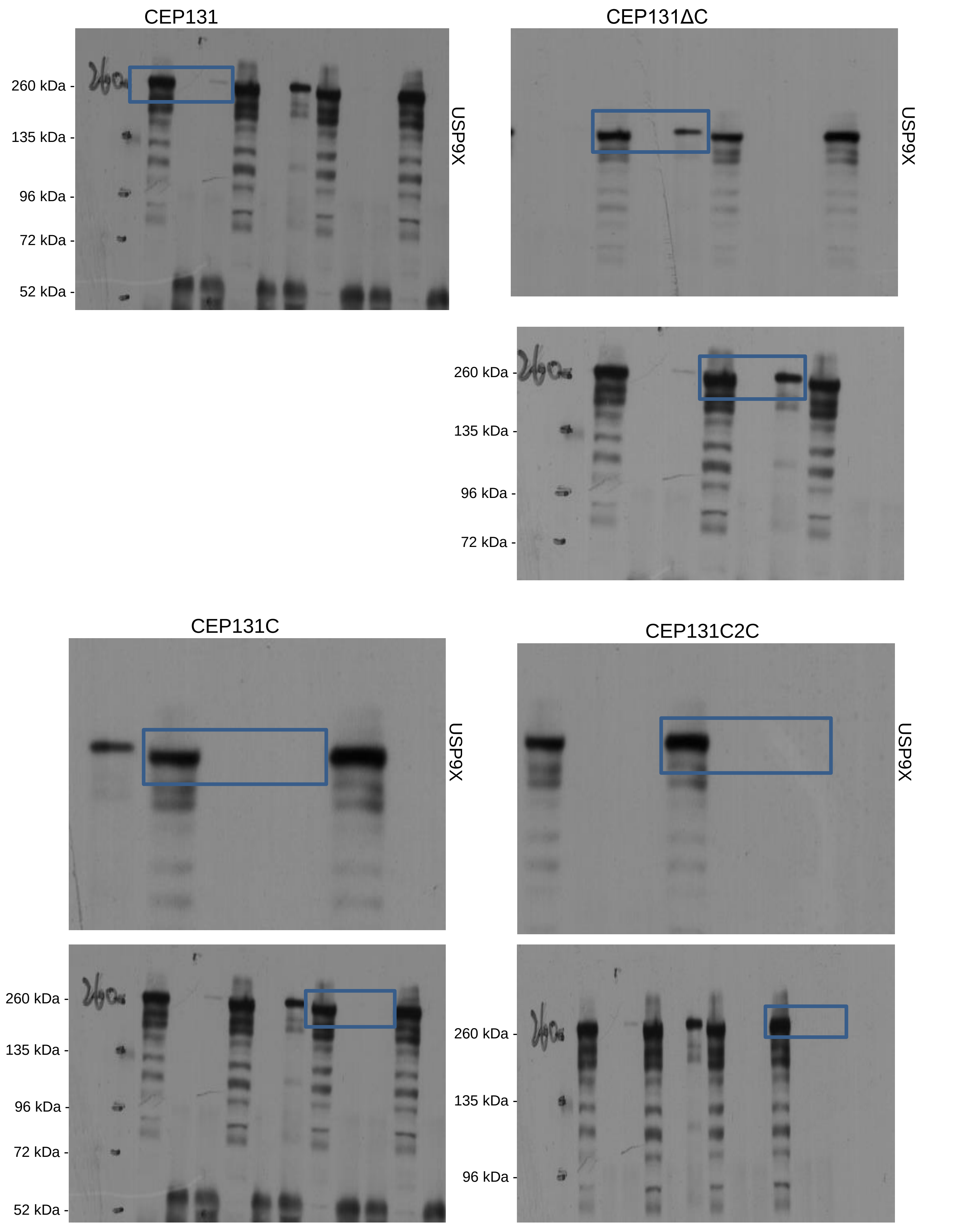

Uncropped blots related to Figure 1e

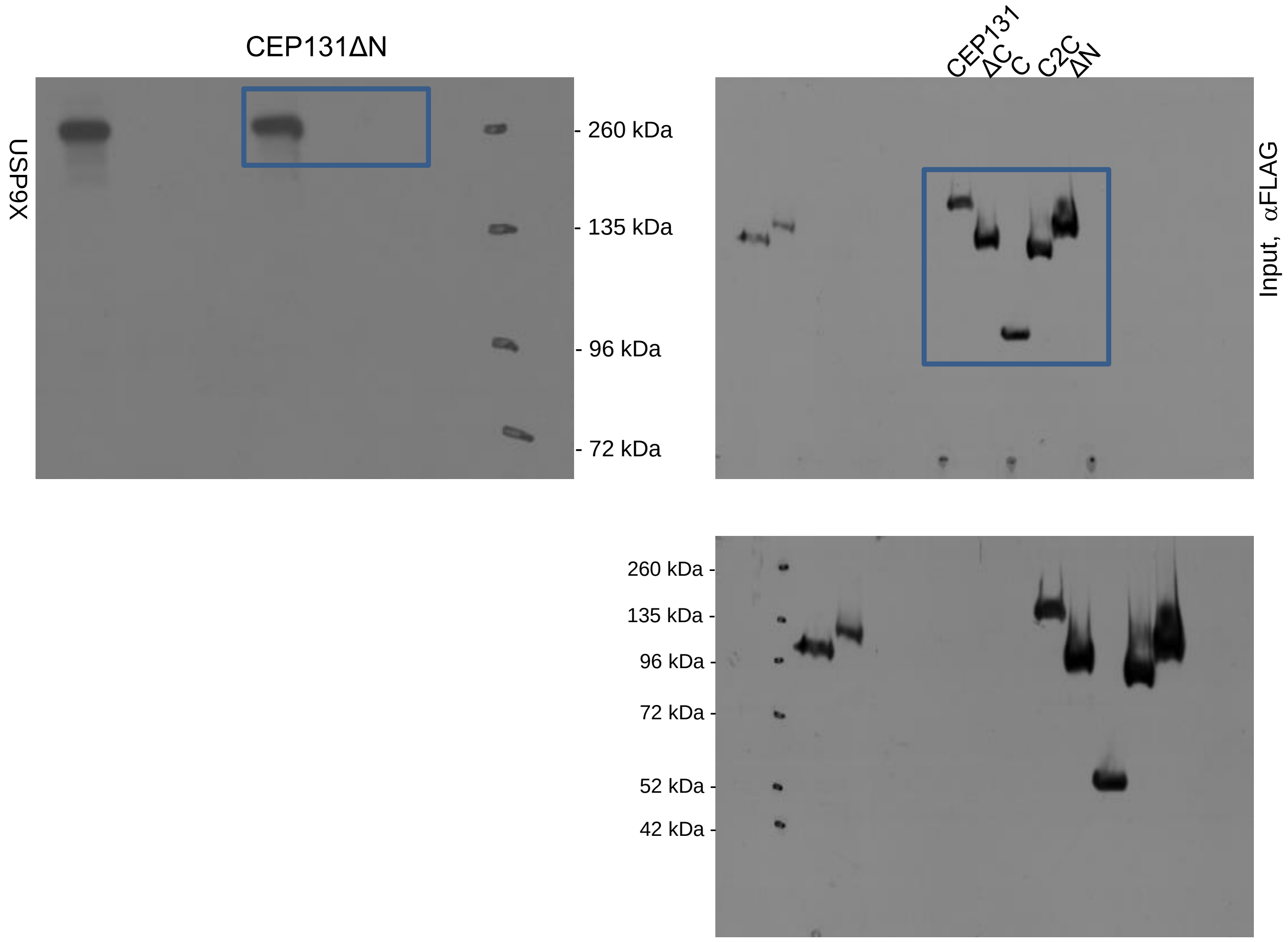

Uncropped blots related to Figure 1e

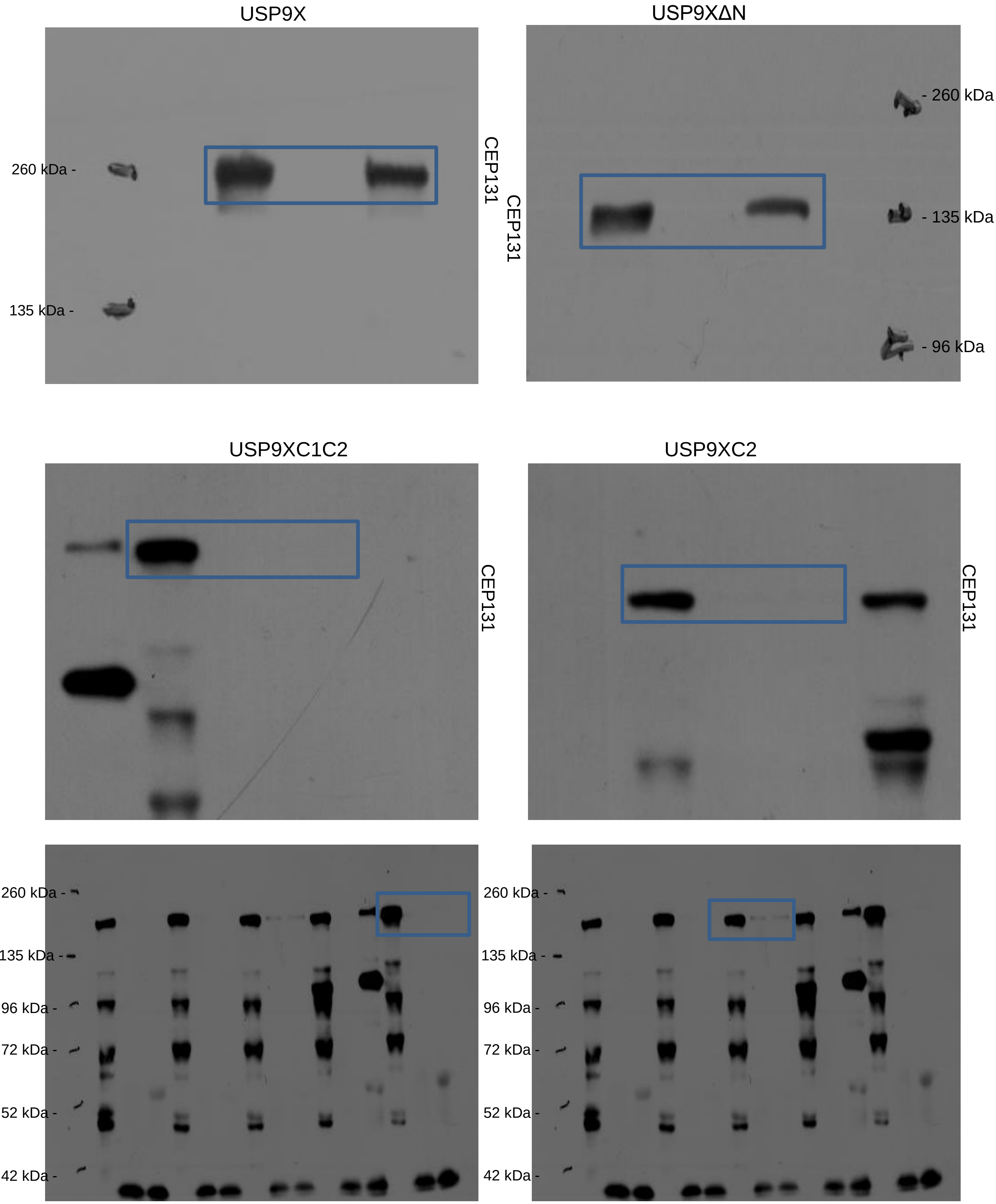

Uncropped blots related to Figure 1e

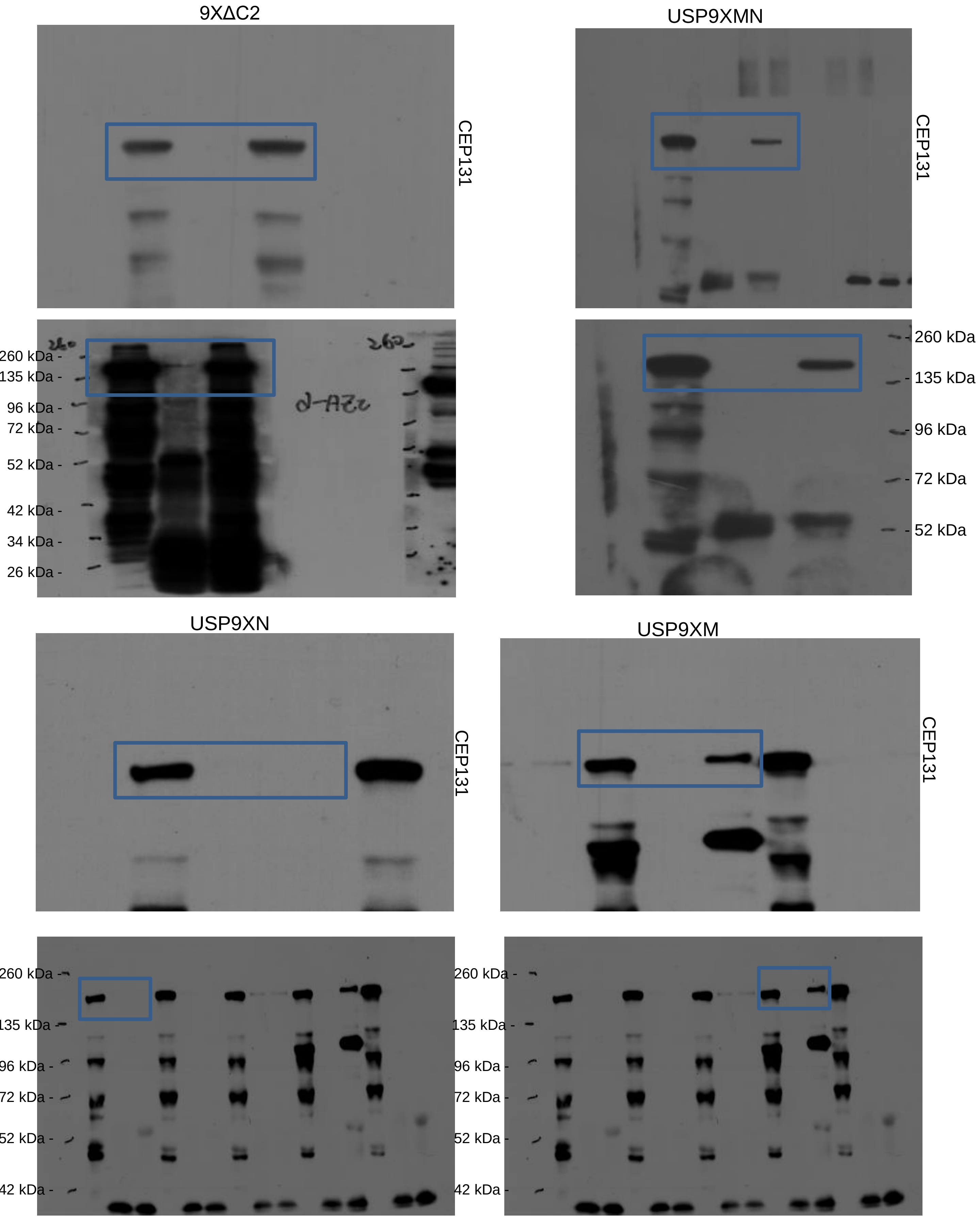

Uncropped blots related to Figure 1e

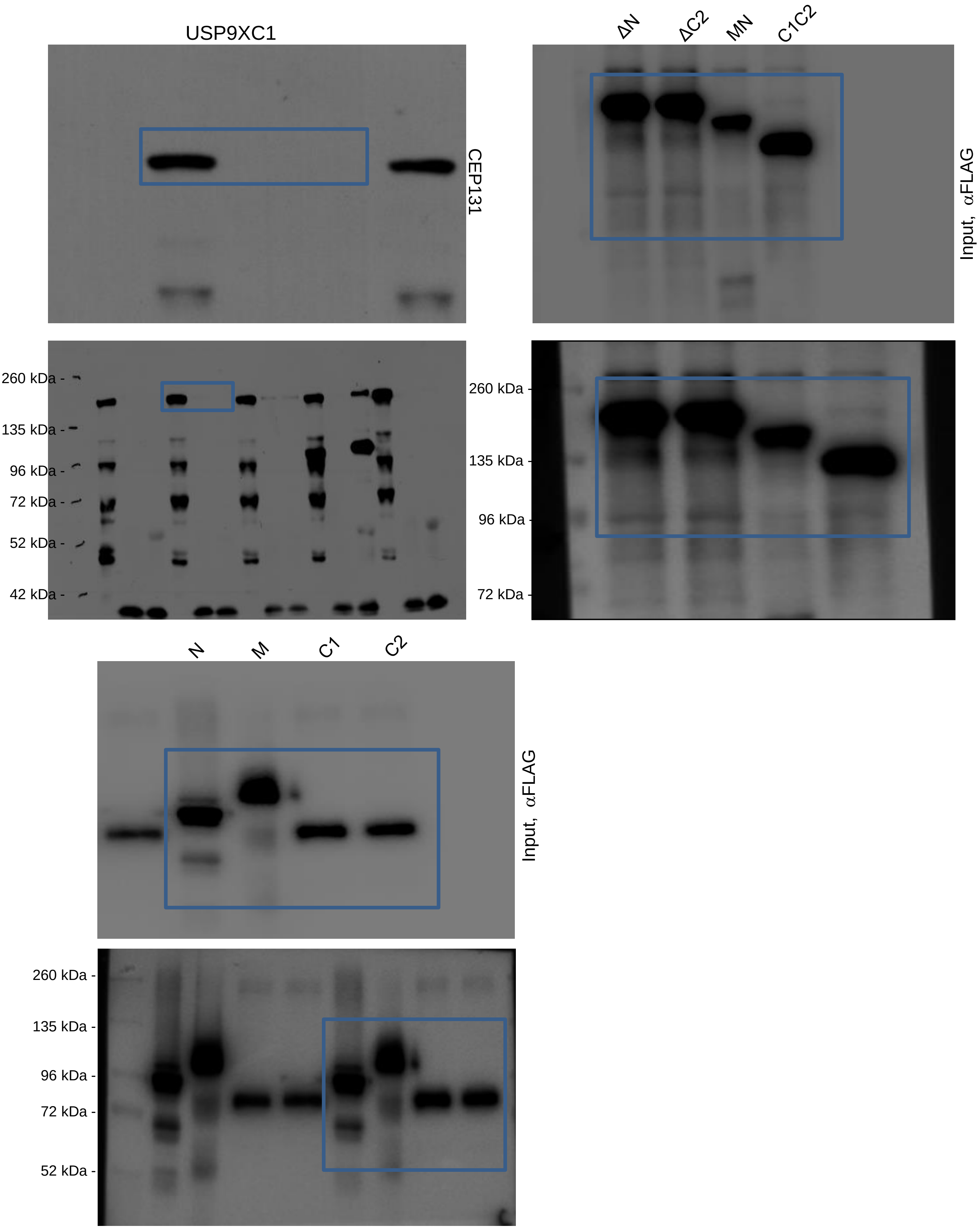

Uncropped blots related to Figure 1f

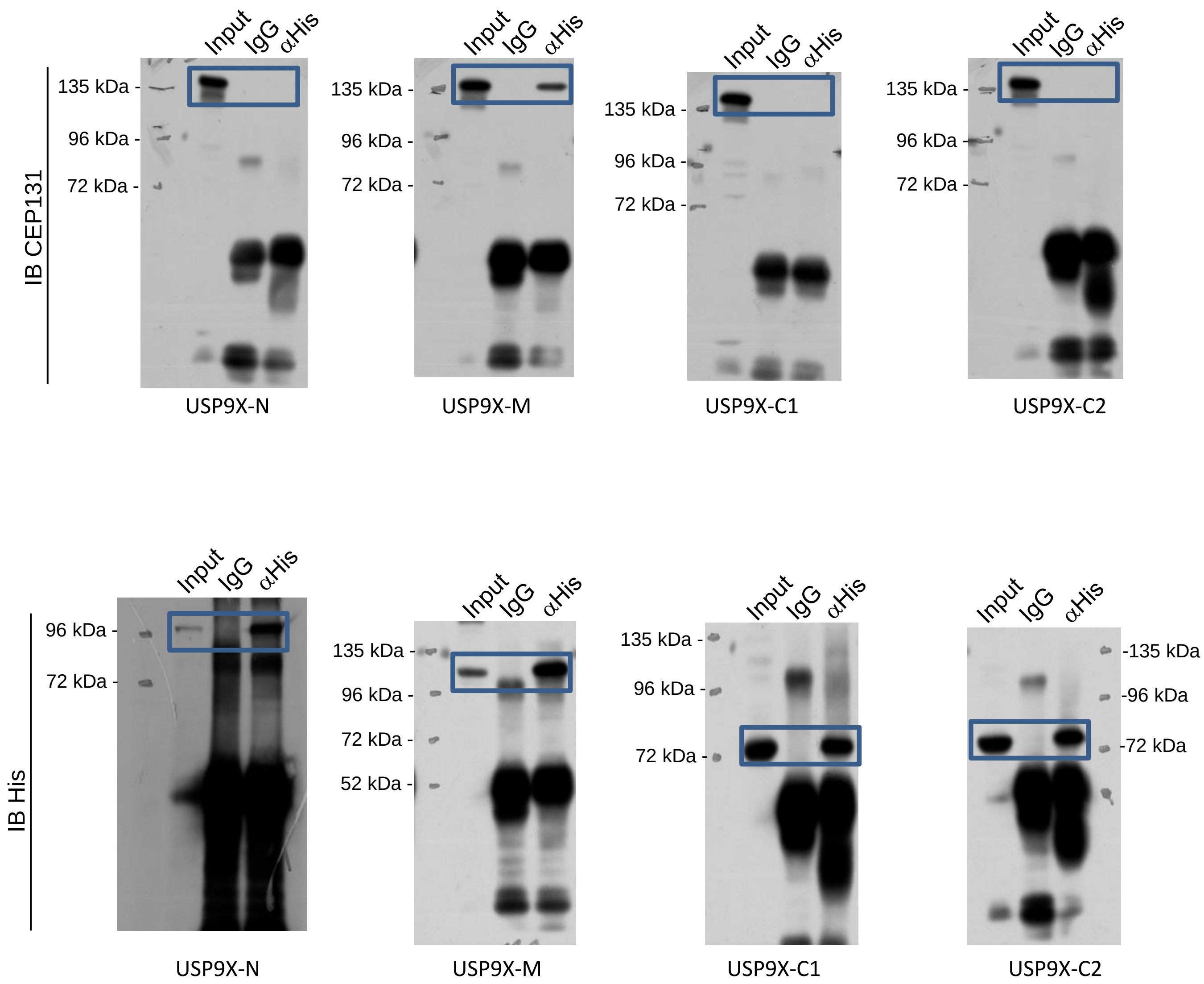

Uncropped blots related to Figure 1g

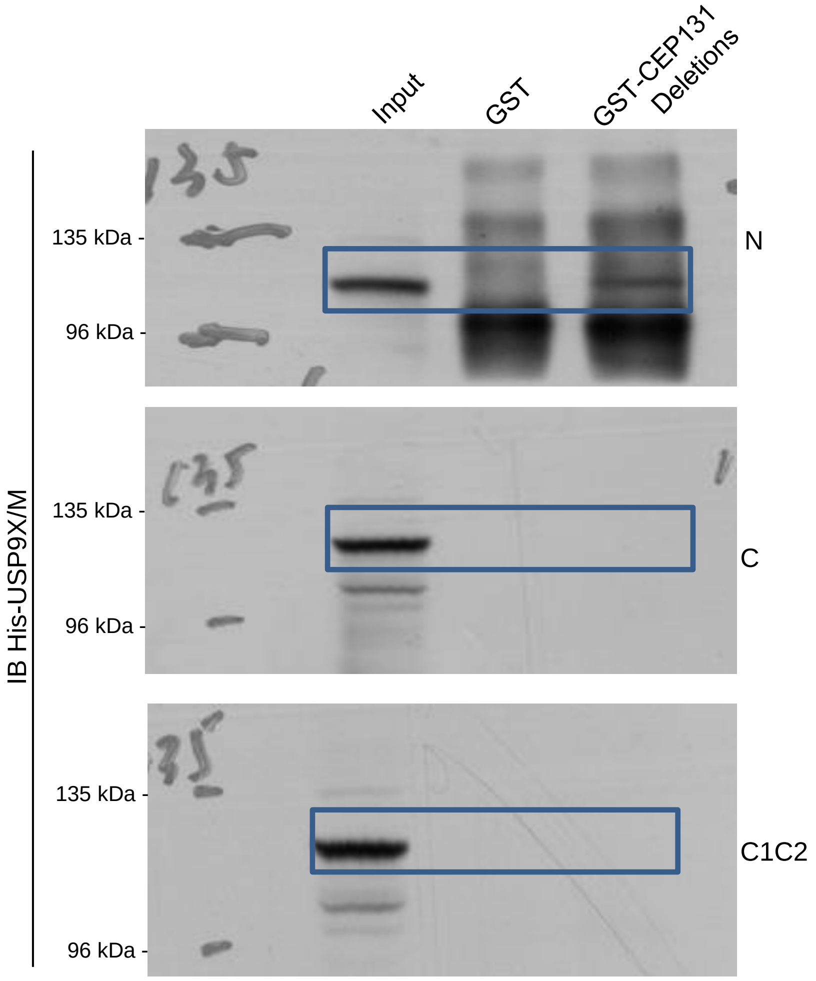

Uncropped blots related to Figure 2d

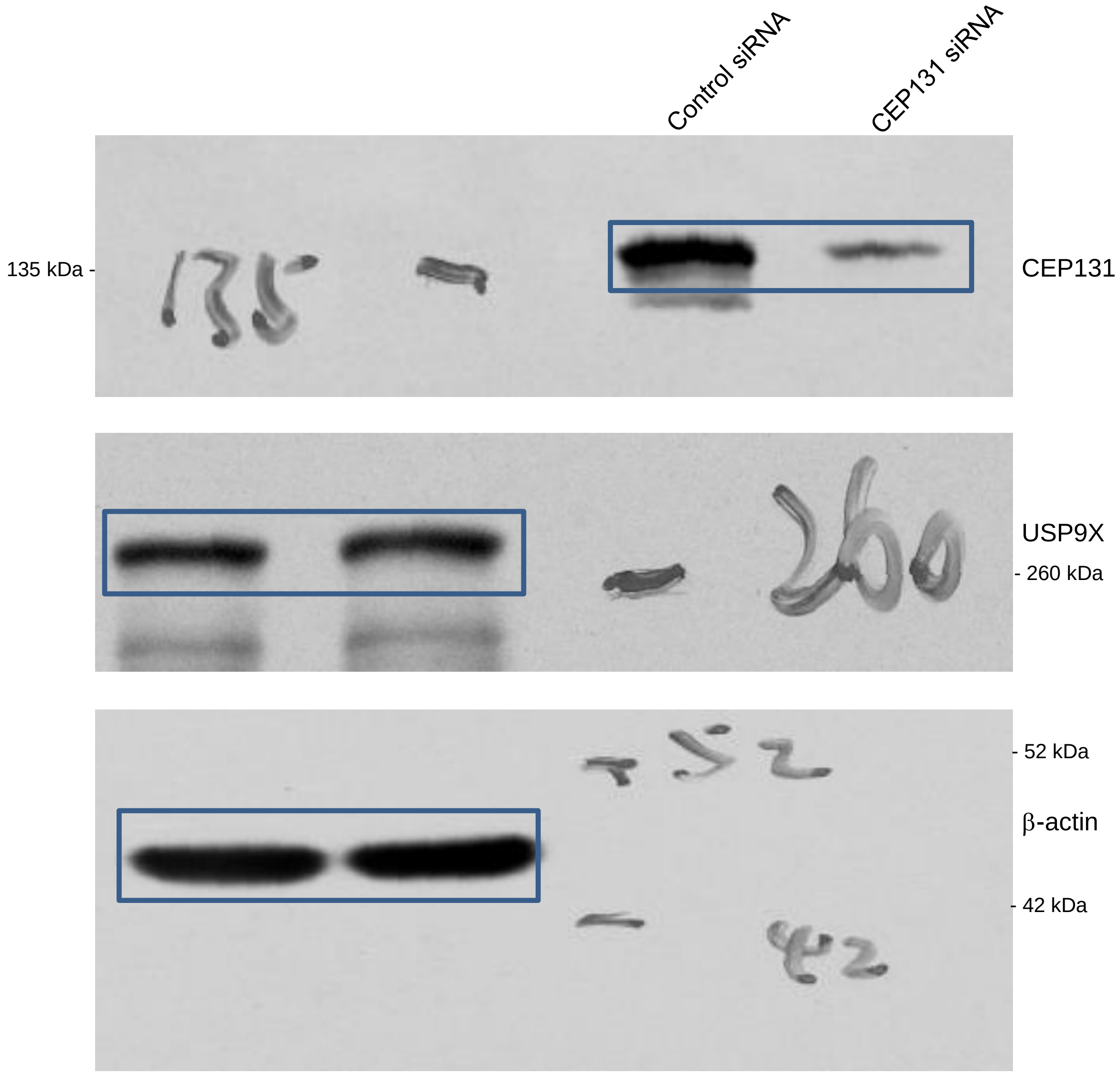

Uncropped blots related to Figure 2e

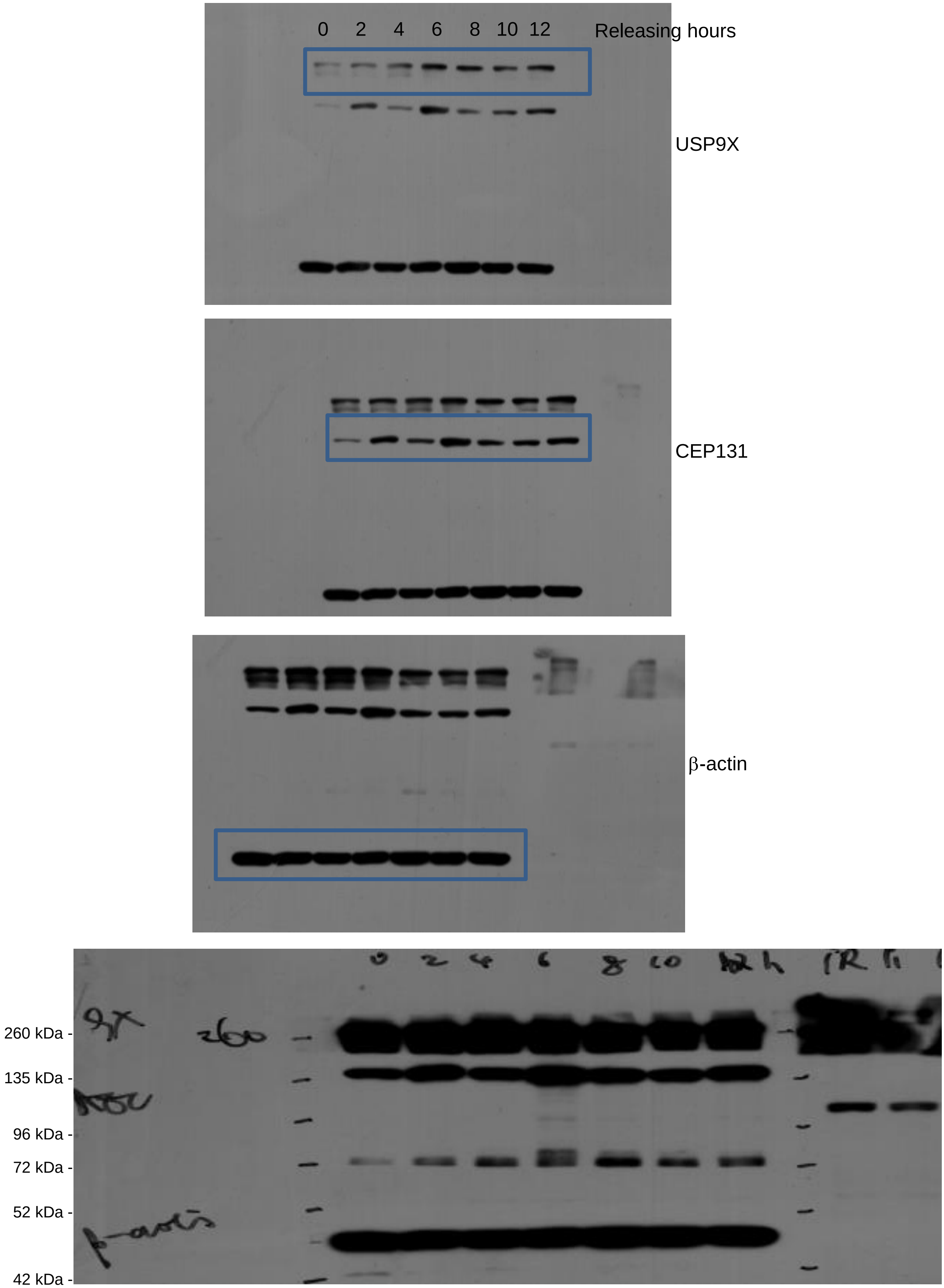

Uncropped blots related to Figure 2e

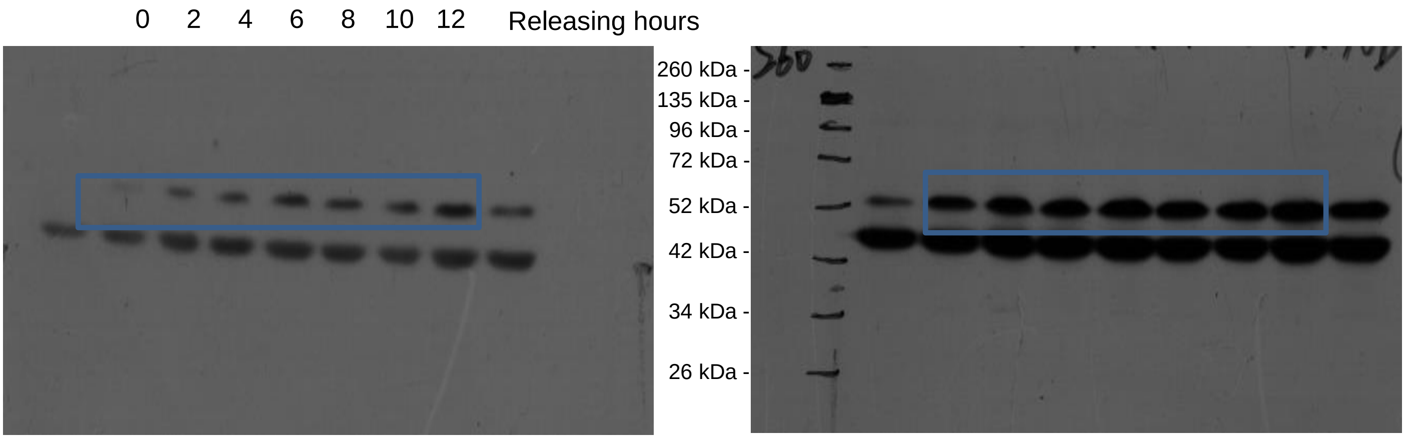

Cyclin A

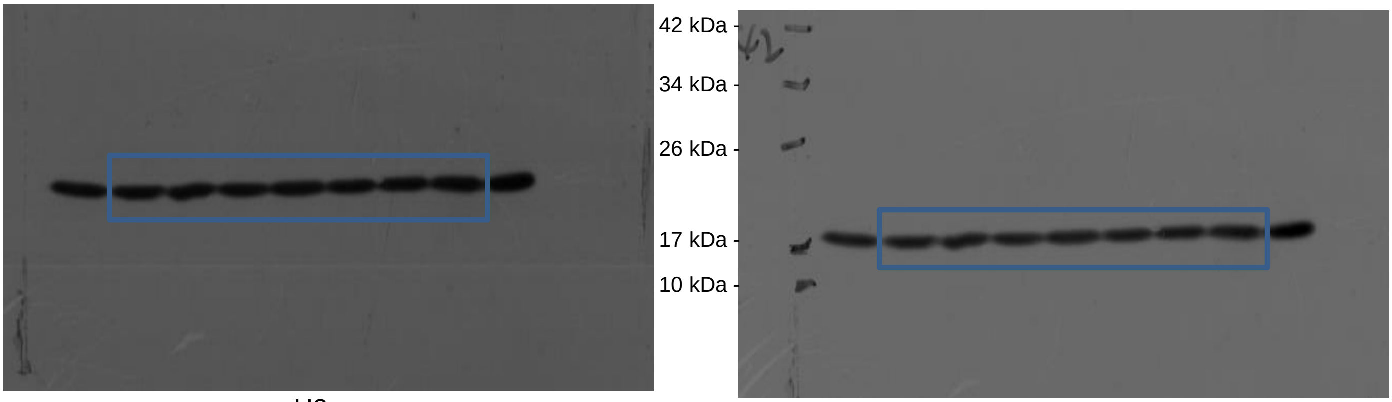

H3

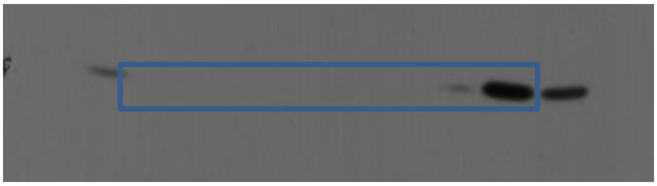

H3S10P

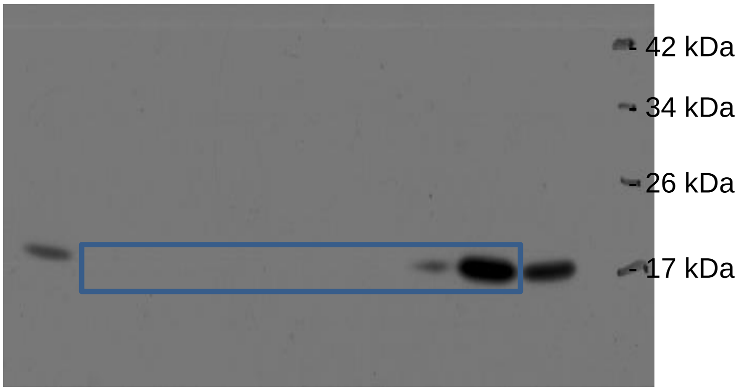

Uncropped blots related to Figure 2f

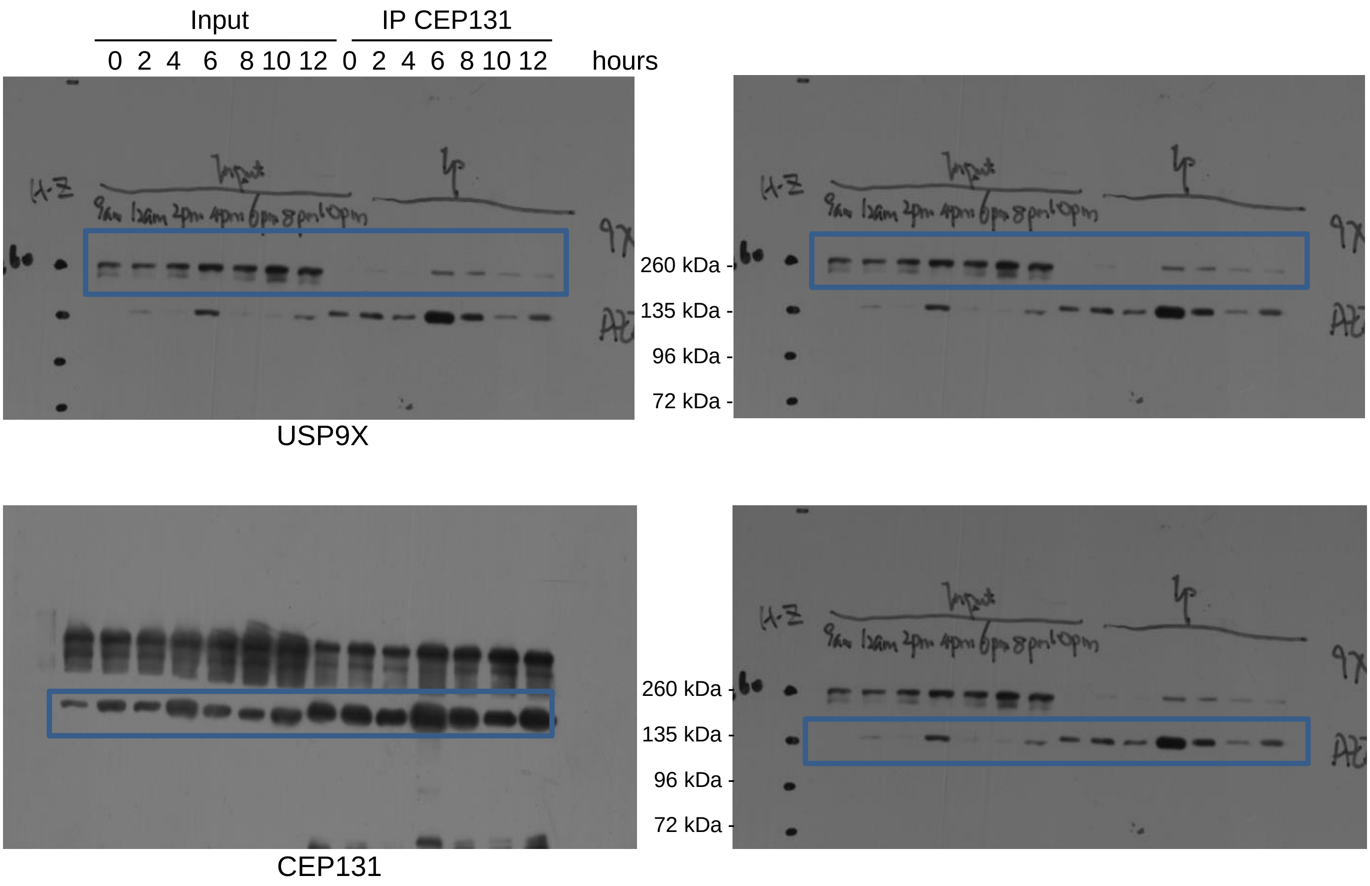

Uncropped blots related to Figure 3a

MCF-7

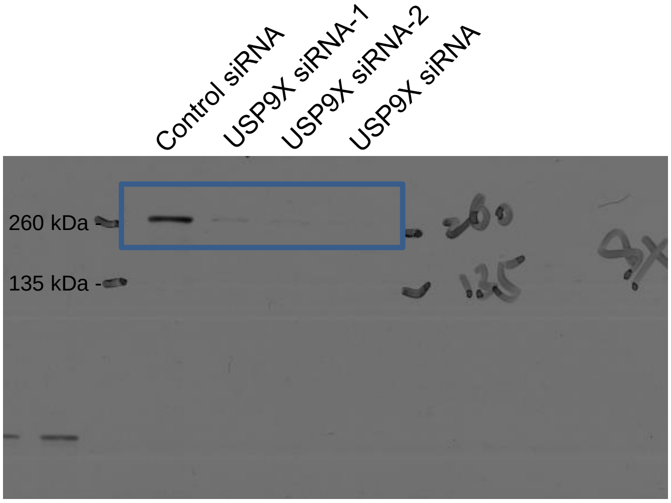

USP9X

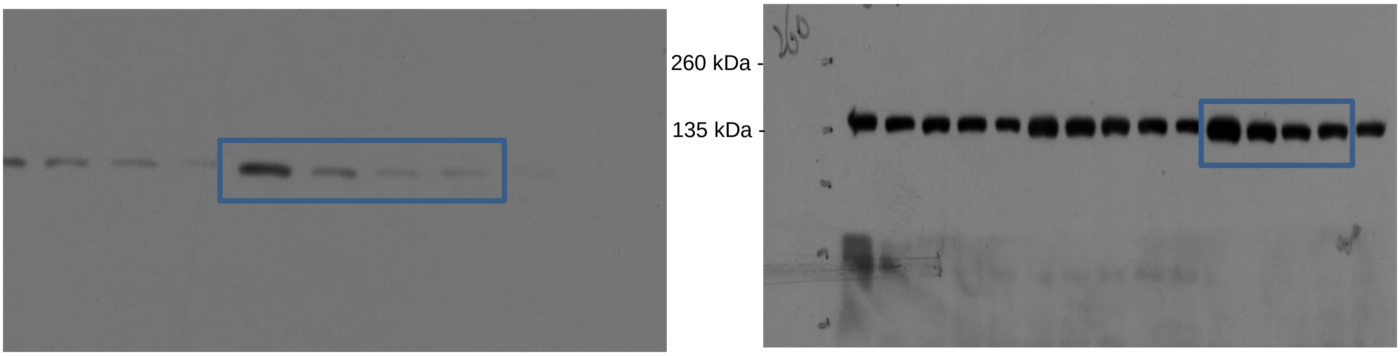

CEP131

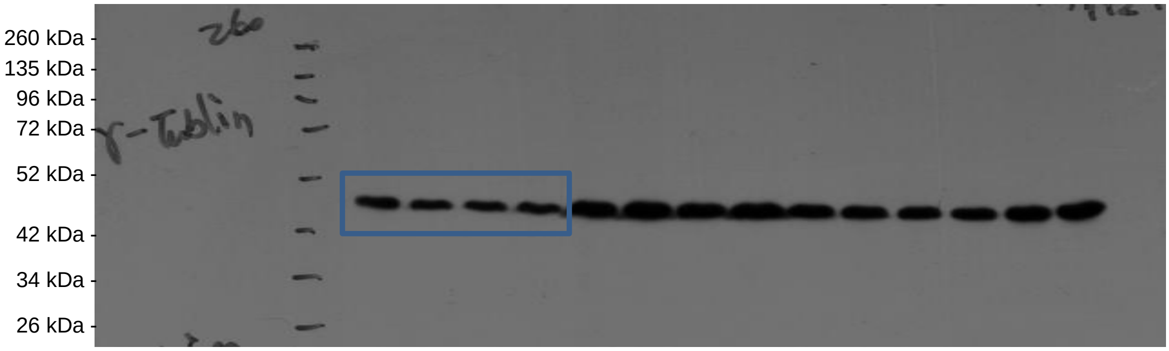

γ-tubulin

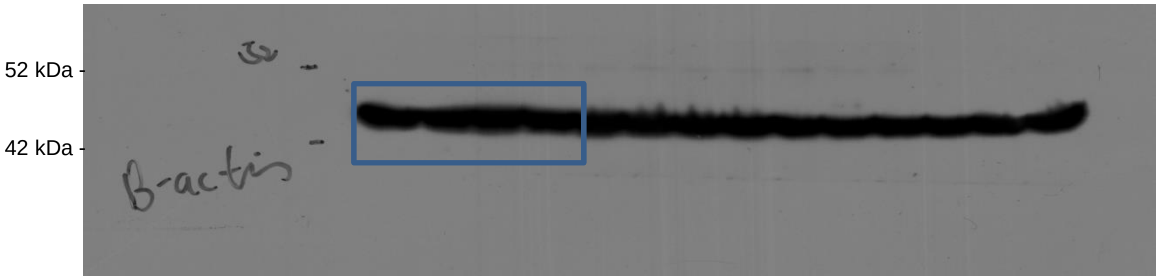

β-actin

Uncropped blots related to Figure 3b

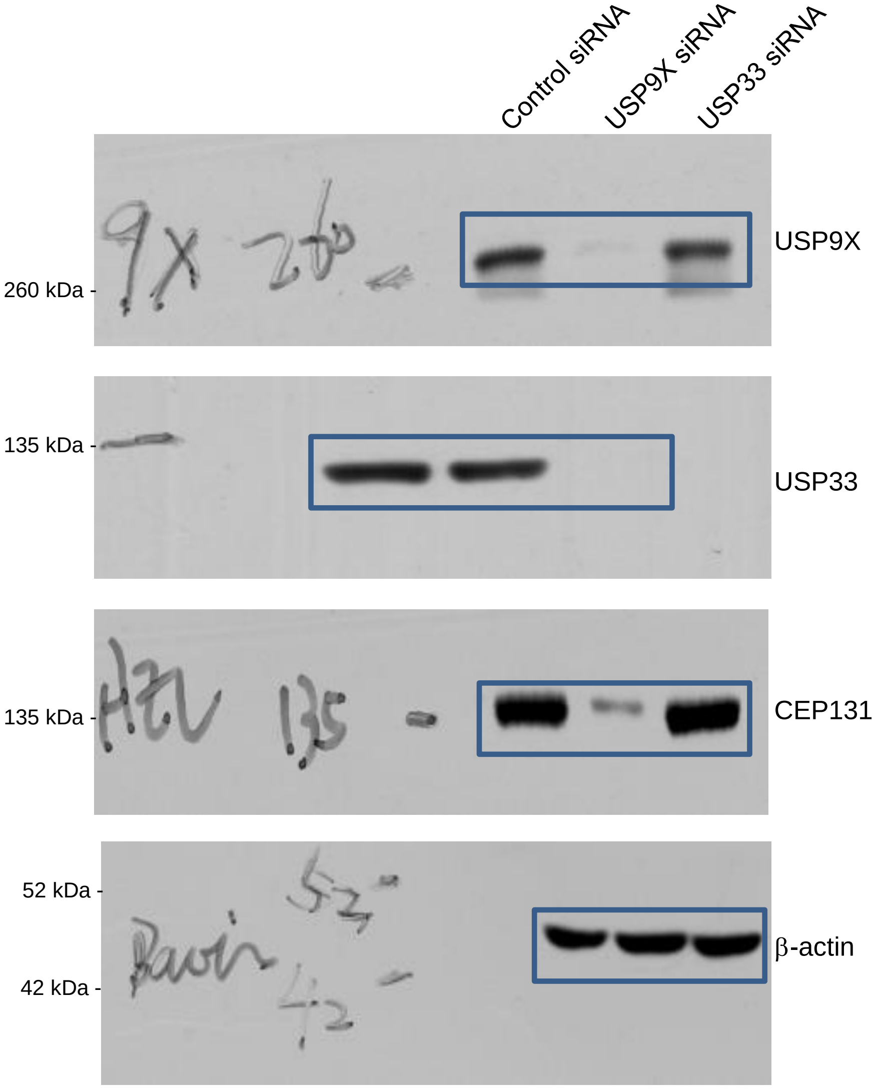

Uncropped blots related to Figure 3c

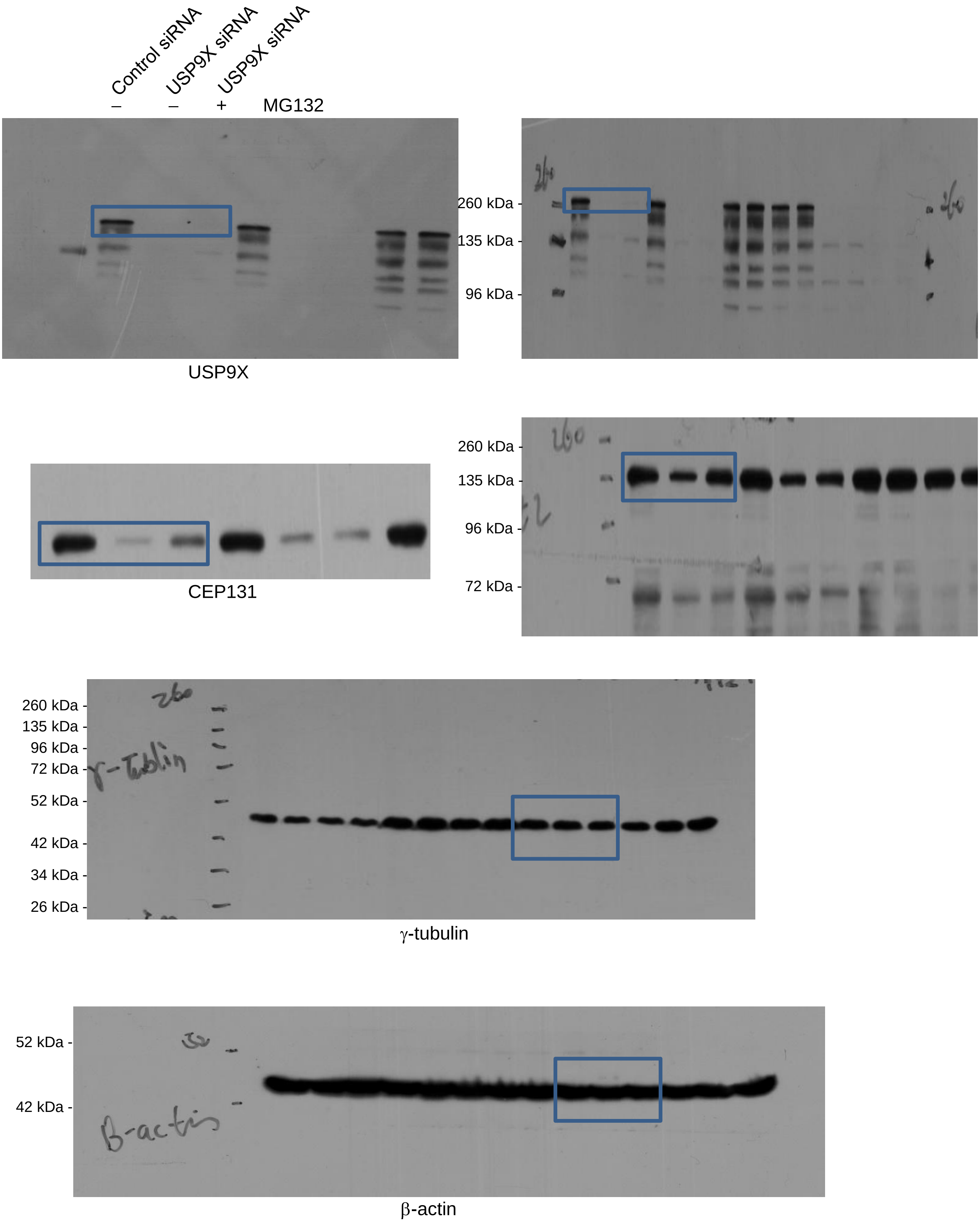

Uncropped blots related to Figure 3d

MCF-7

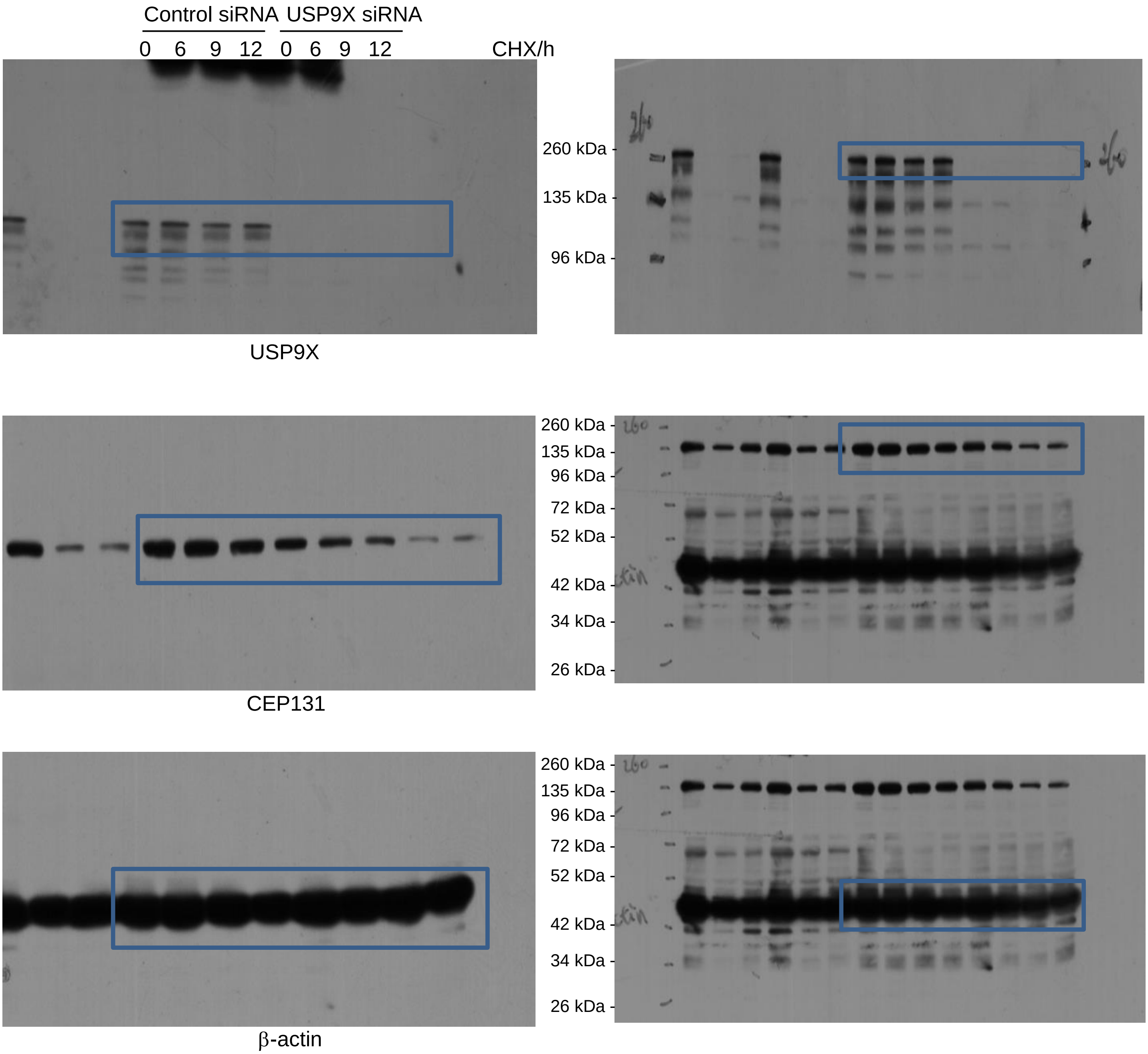

Uncropped blots related to Figure 3e

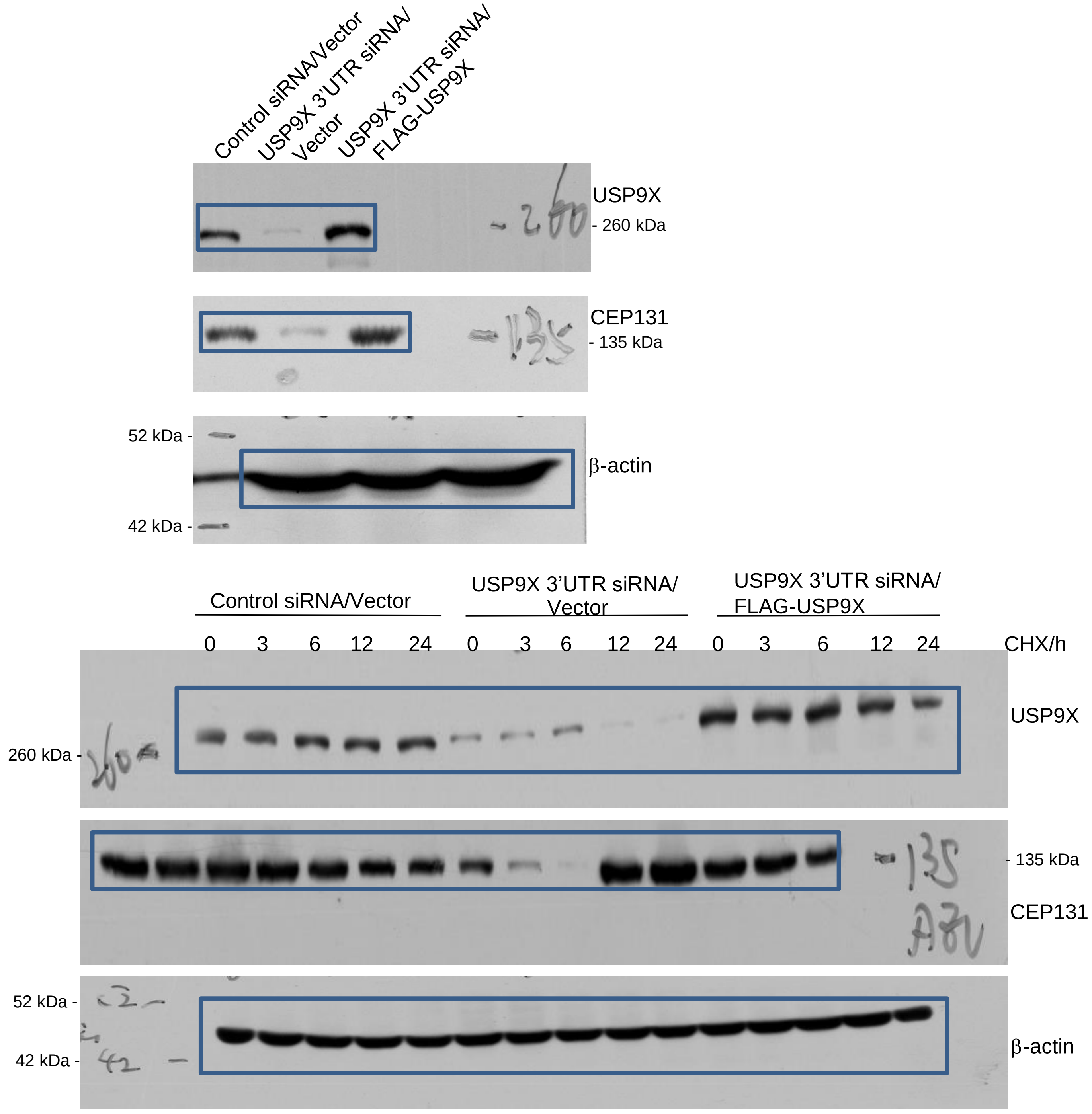

Uncropped blots related to Figure 3f

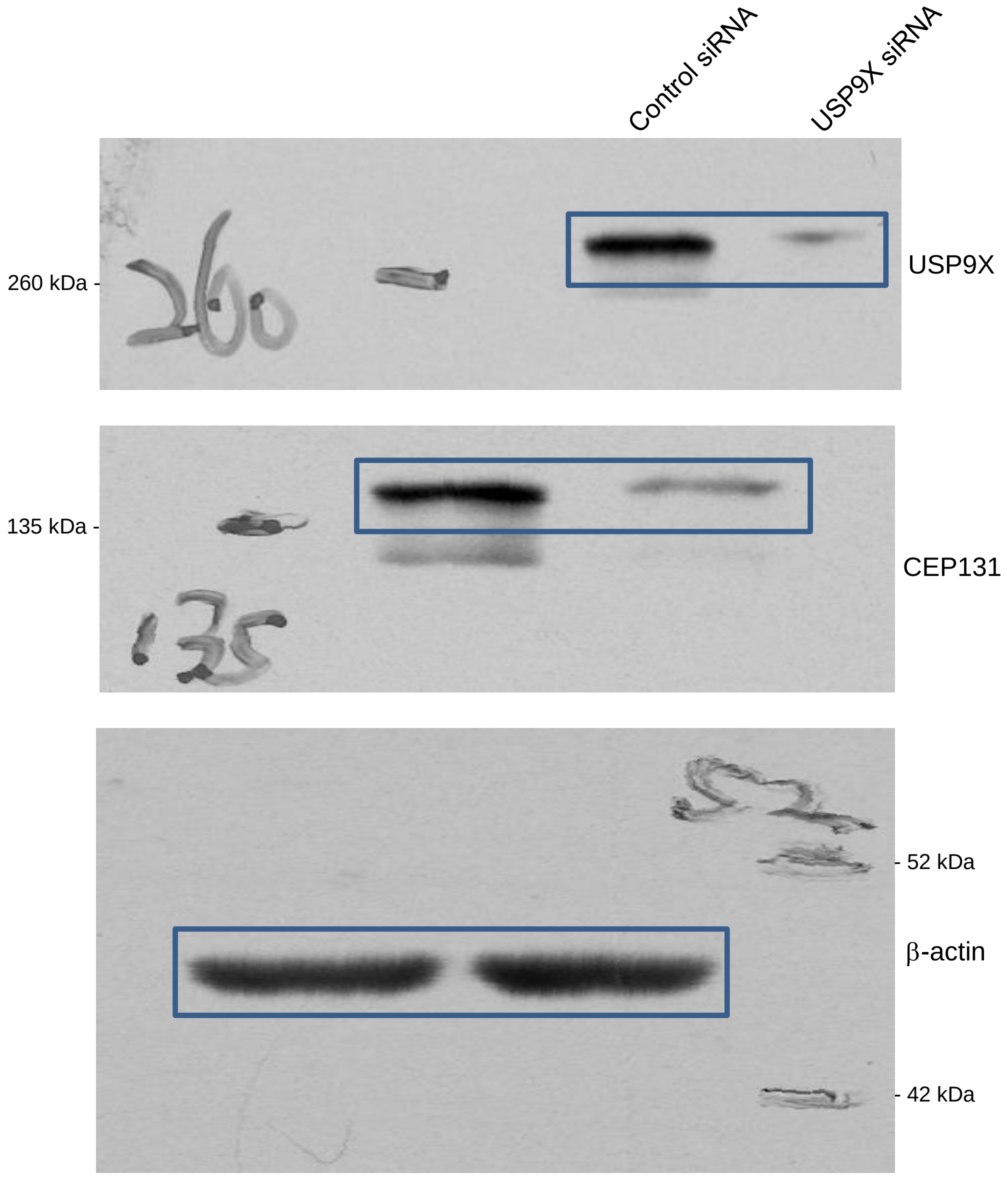

Uncropped blots related to Figure 3g

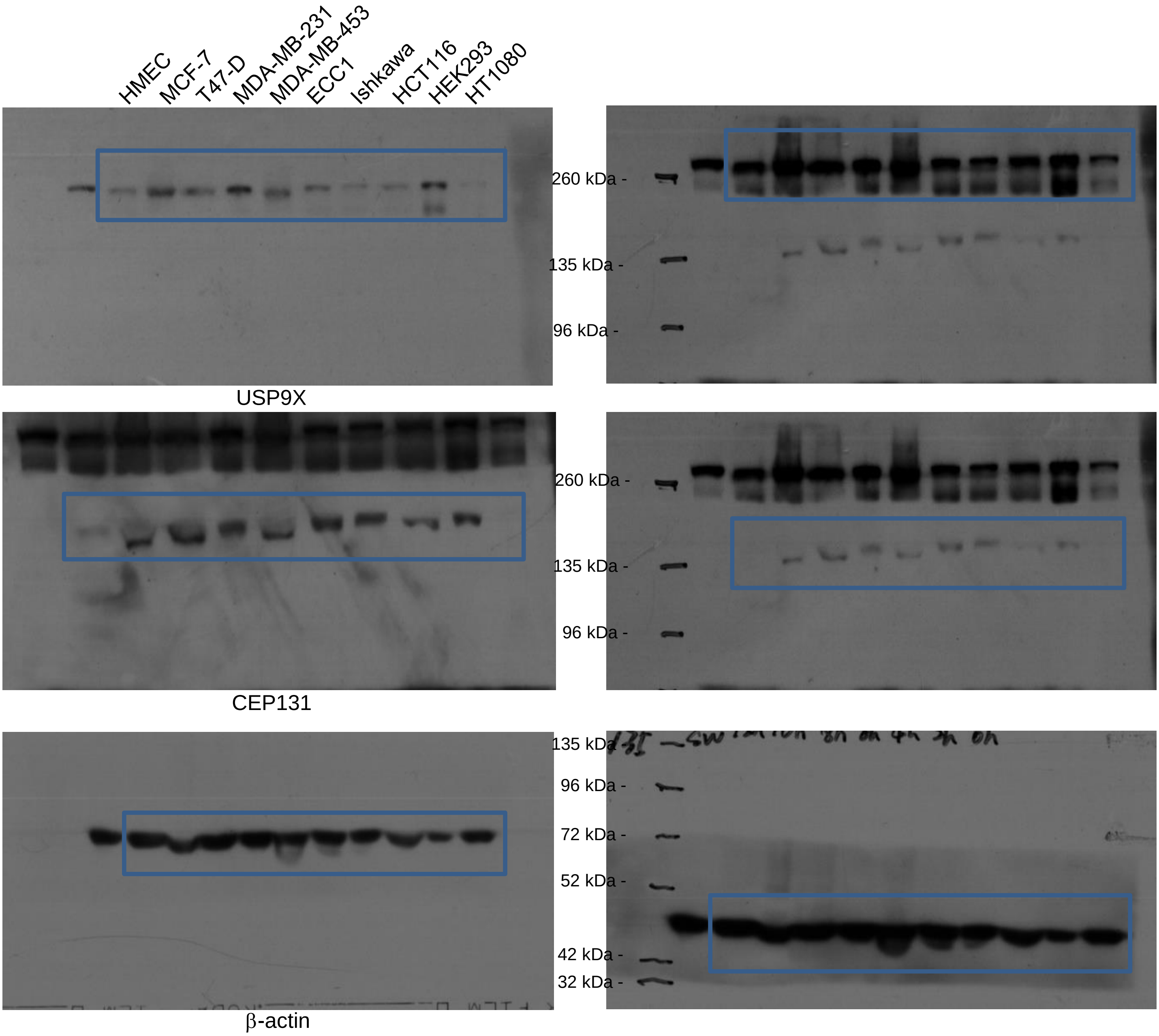

Uncropped blots related to Figure 4a

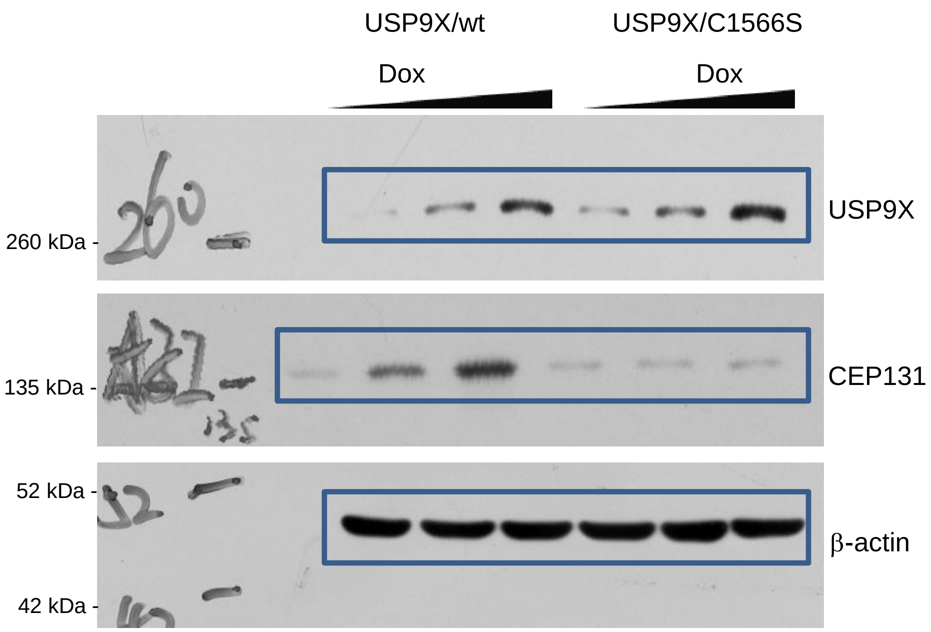

Uncropped blots related to Figure 4b

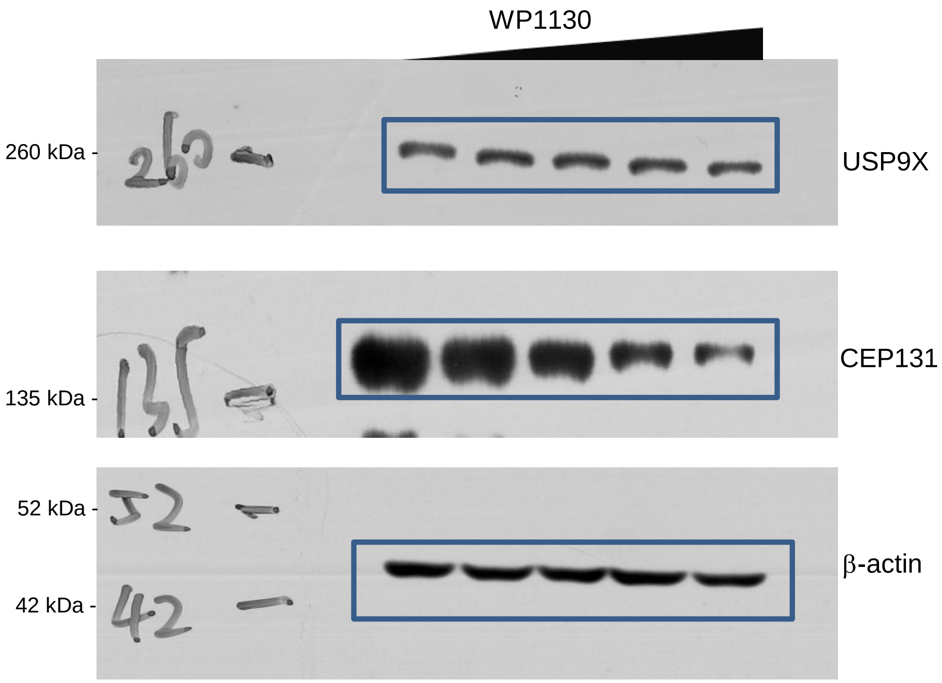

Uncropped blots related to Figure 4c

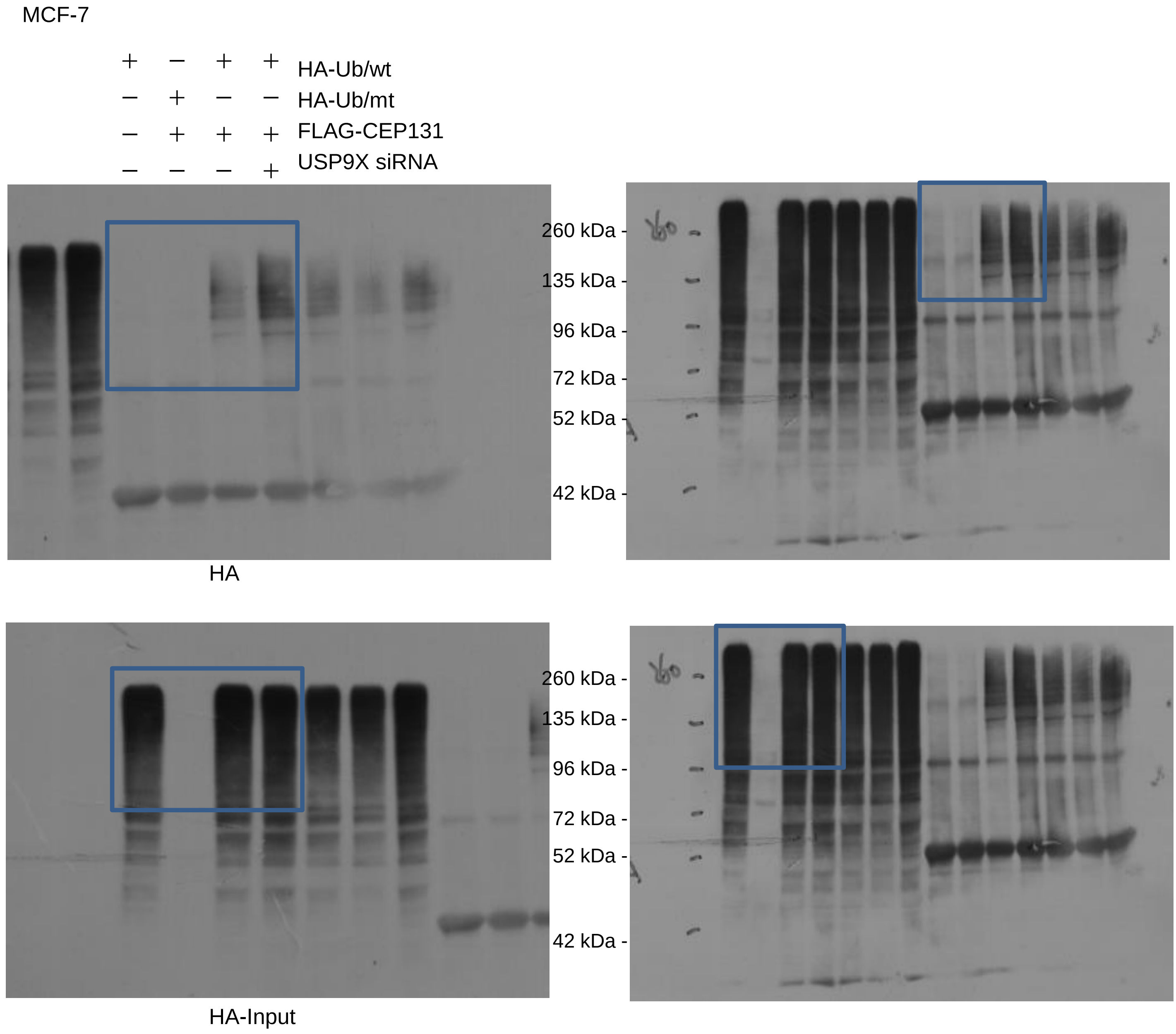

Uncropped blots related to Figure 4c

MCF-7

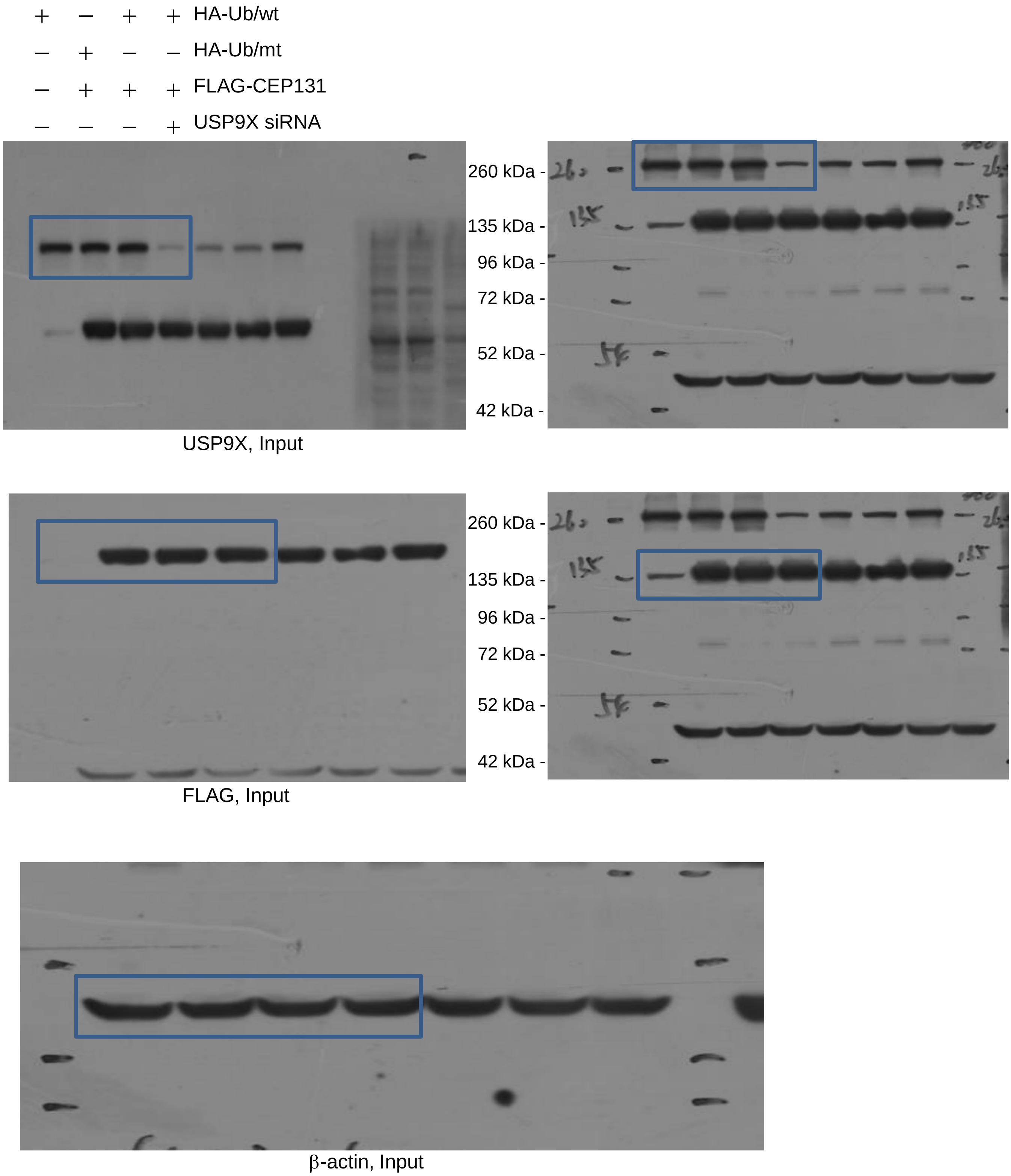

Uncropped blots related to Figure 4d

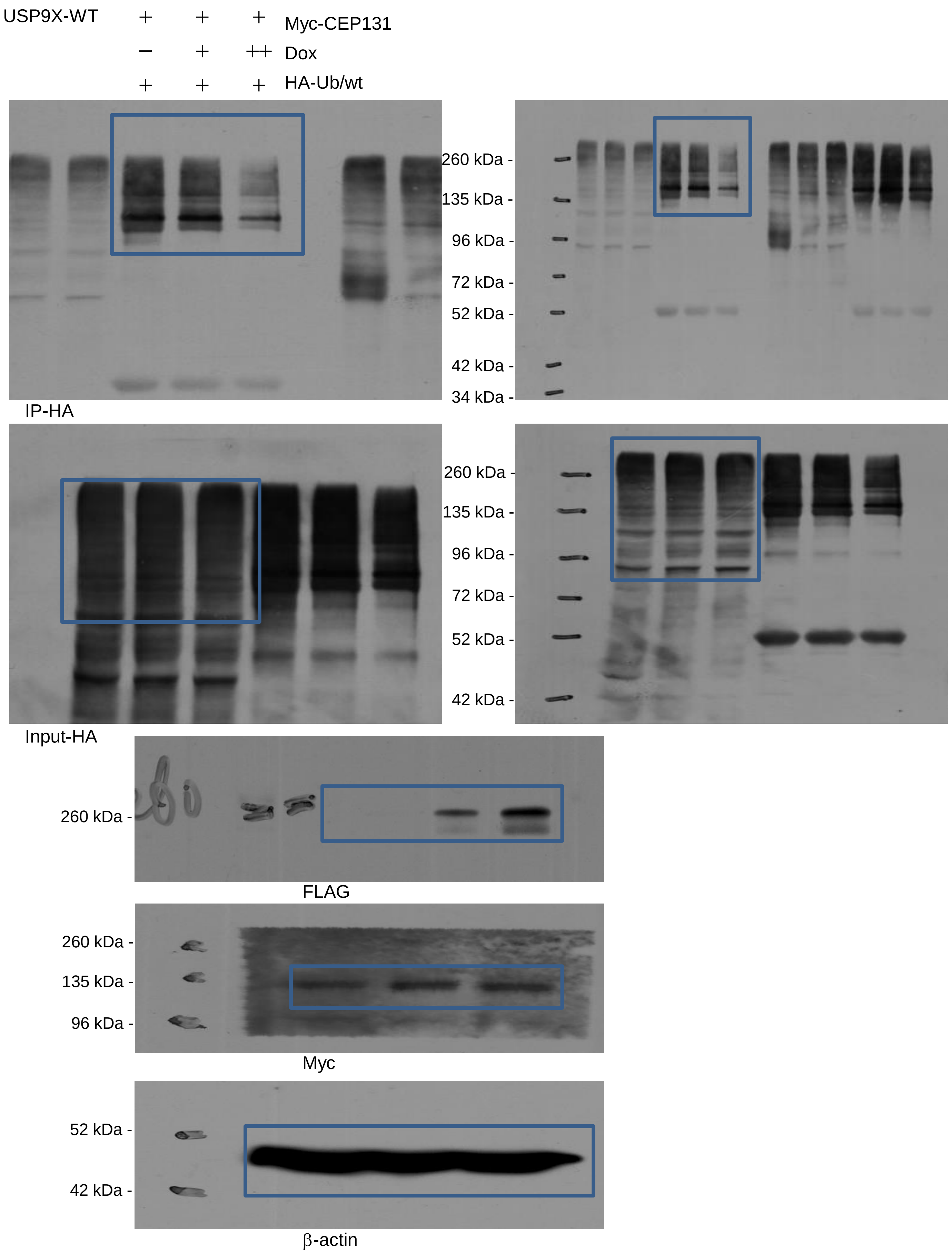

Uncropped blots related to Figure 4e

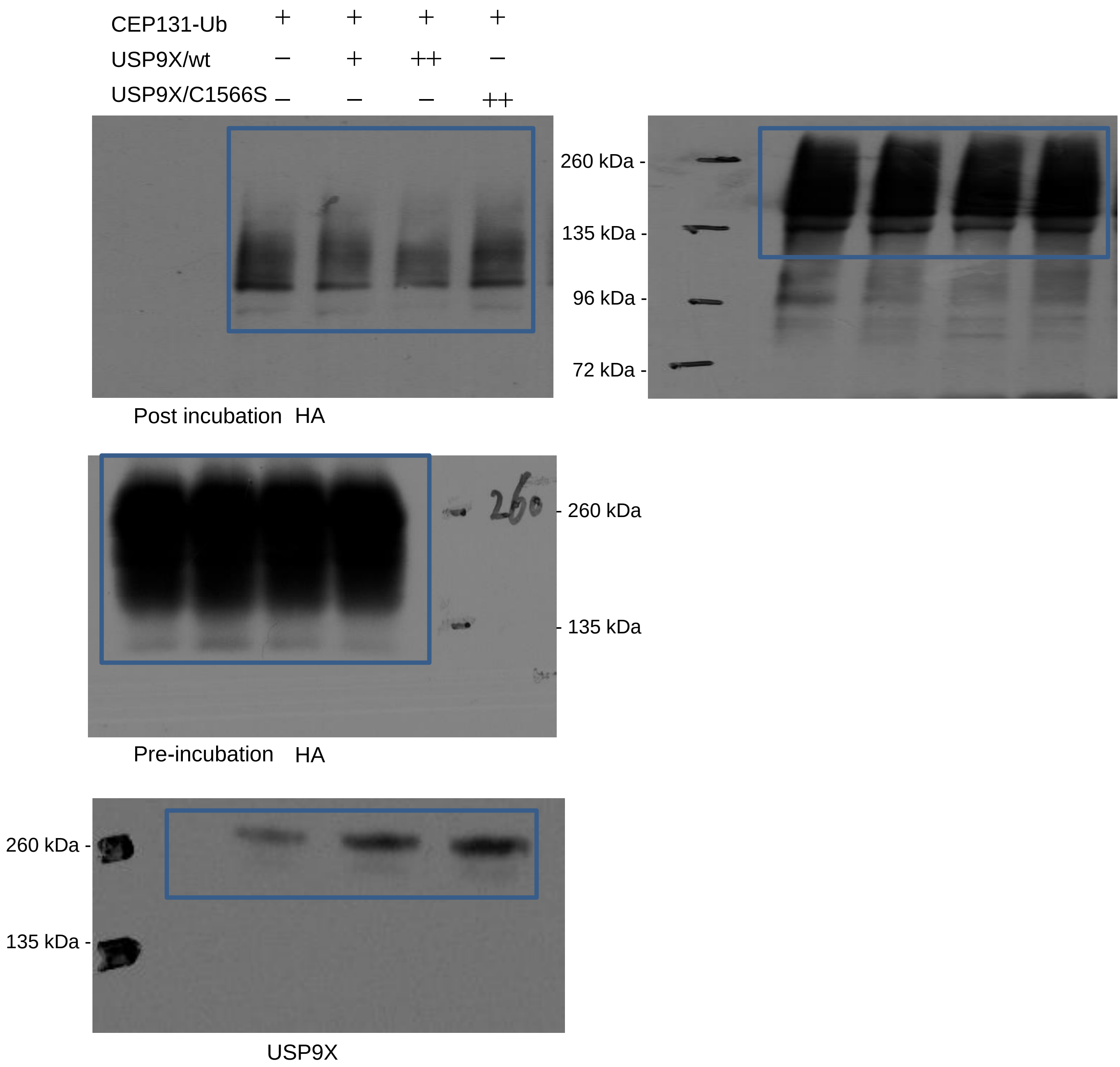

Uncropped blots related to Figure 4f

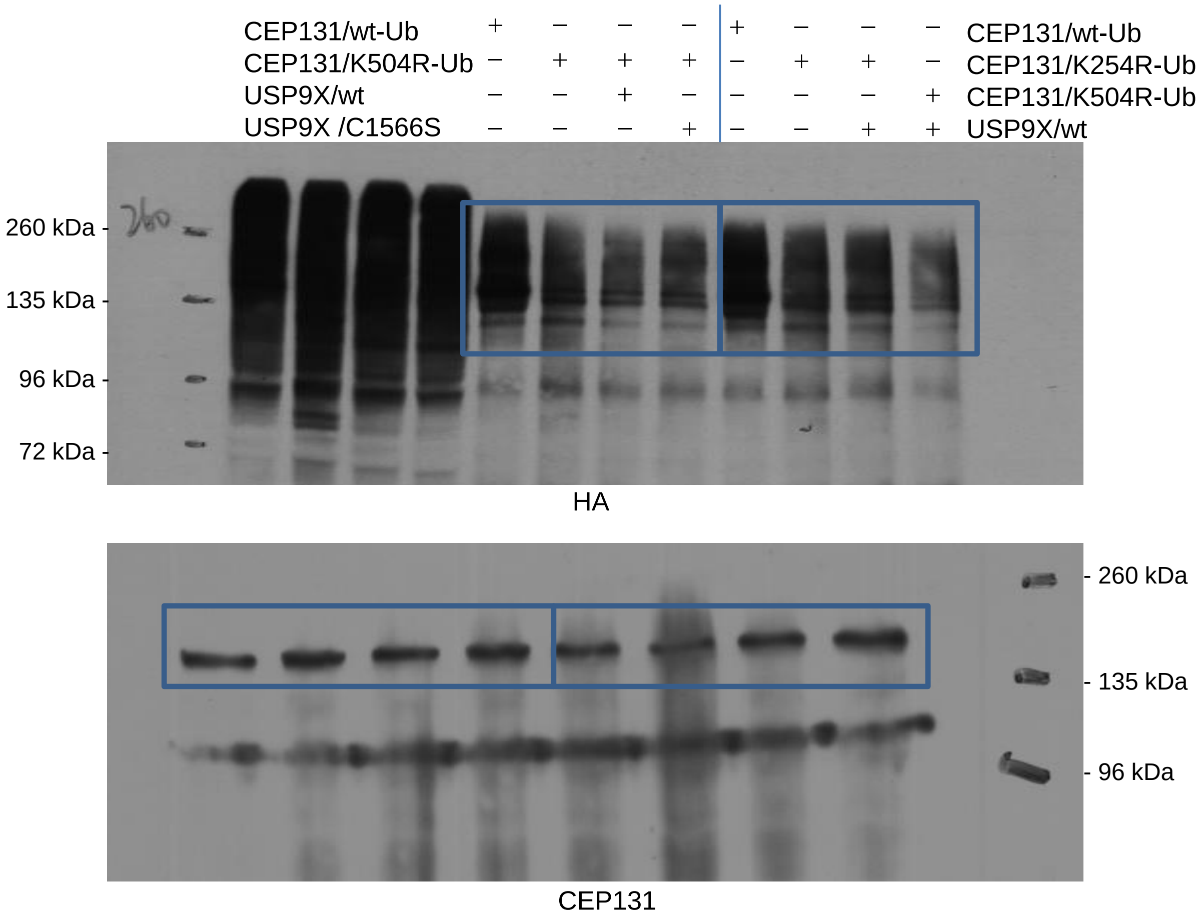

Uncropped blots related to Figure 4g

CEP131/wt

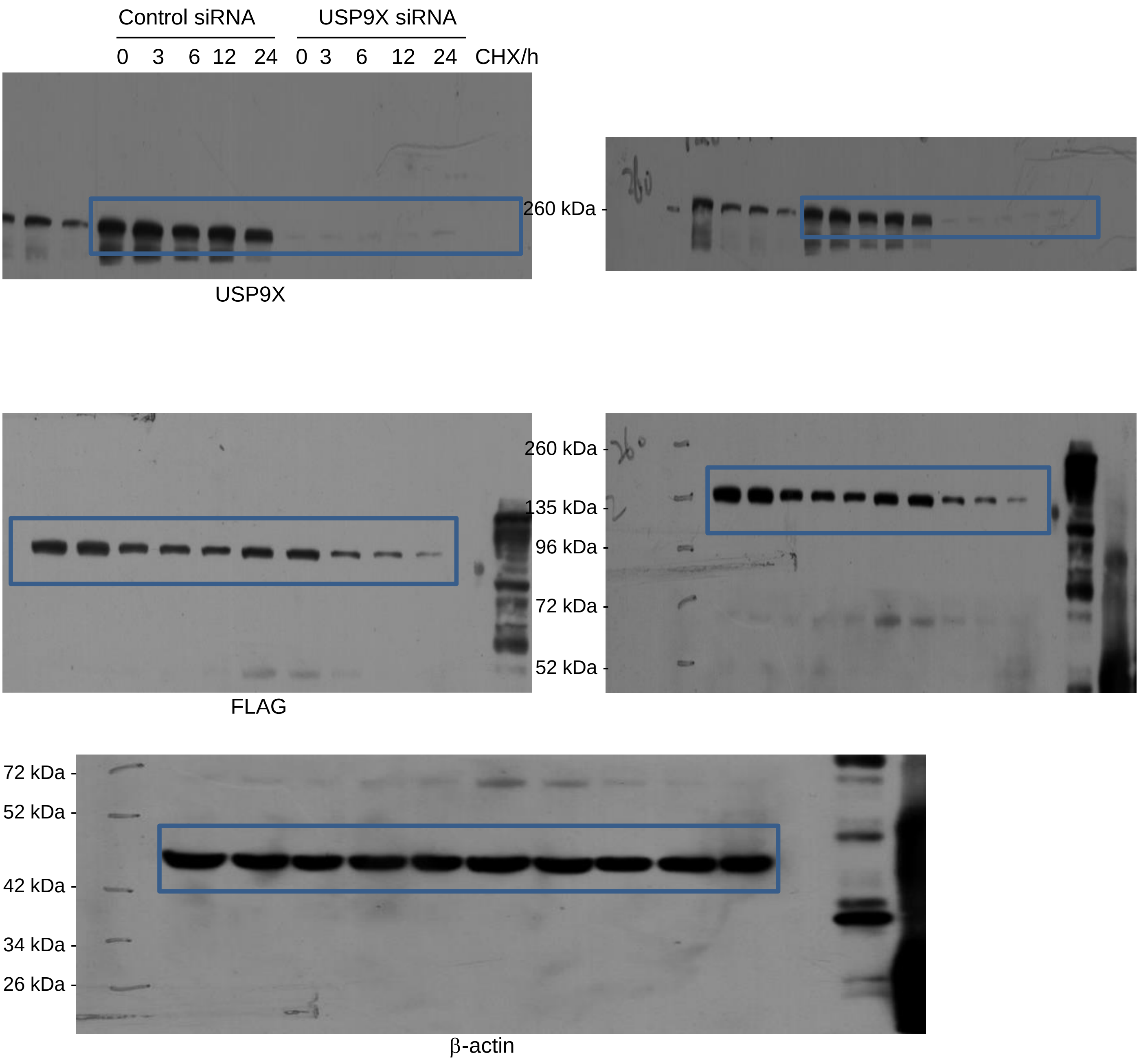

Uncropped blots related to Figure 4g

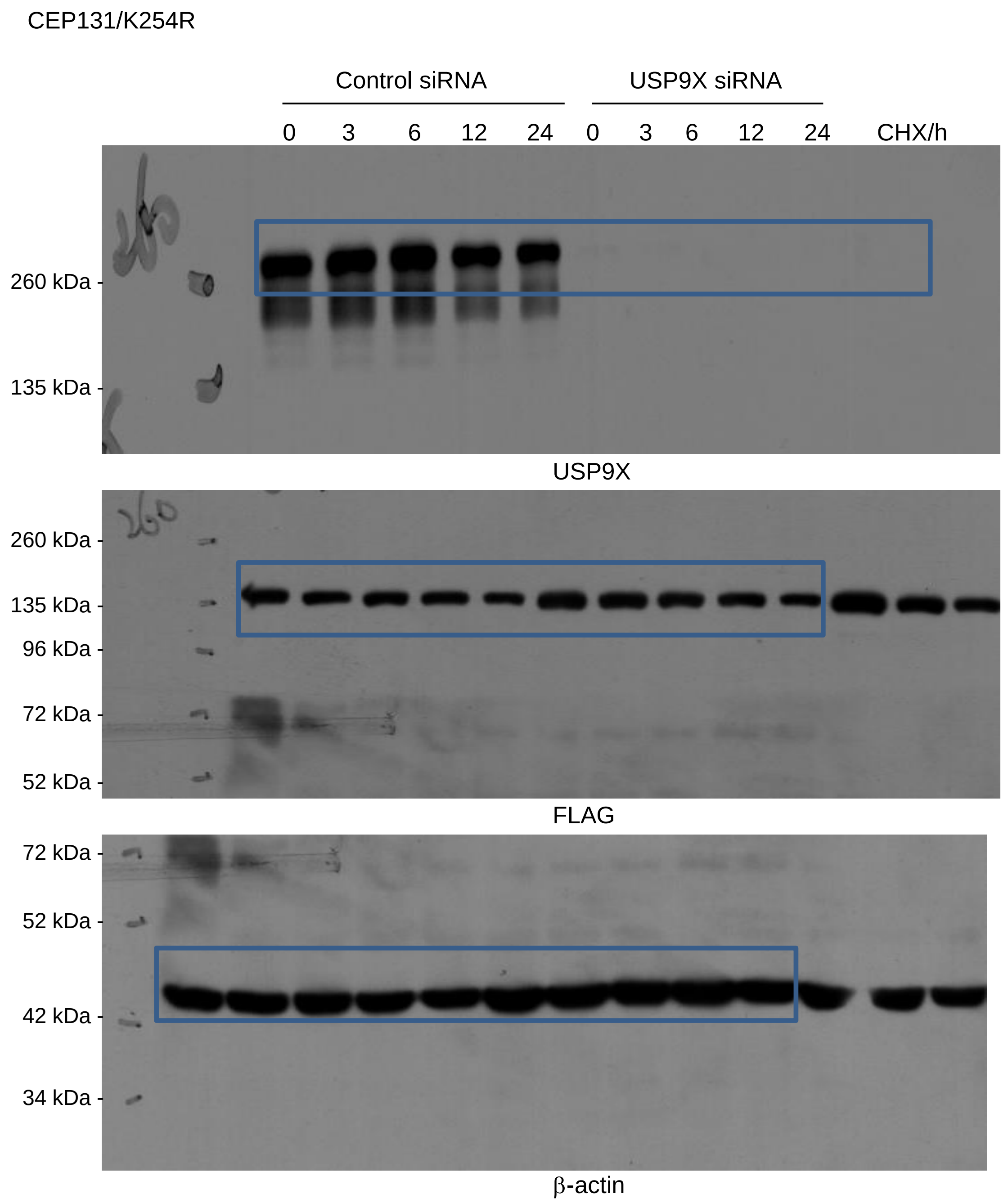

Uncropped blots related to Figure 4h

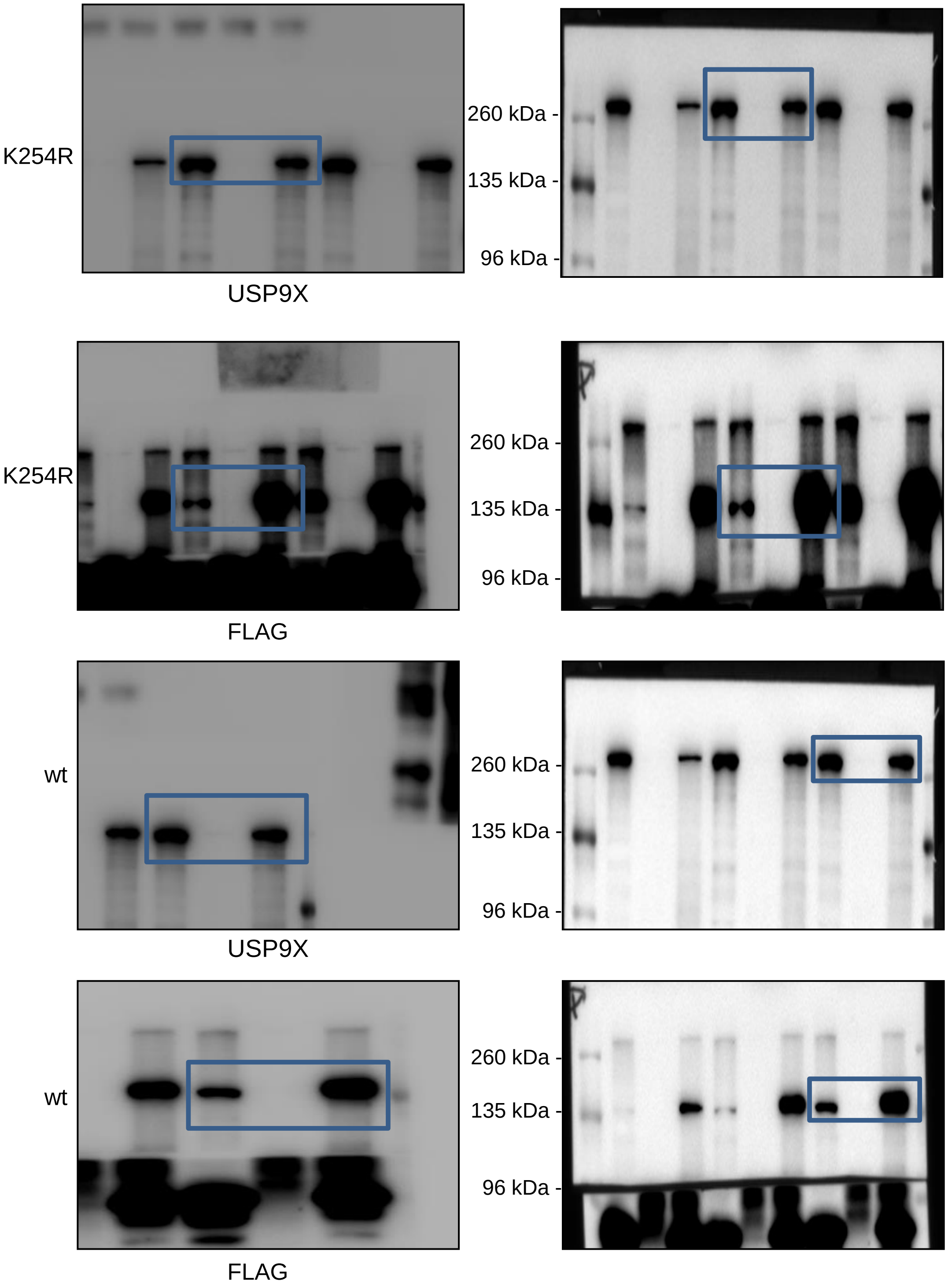

Uncropped blots related to Figure 5b

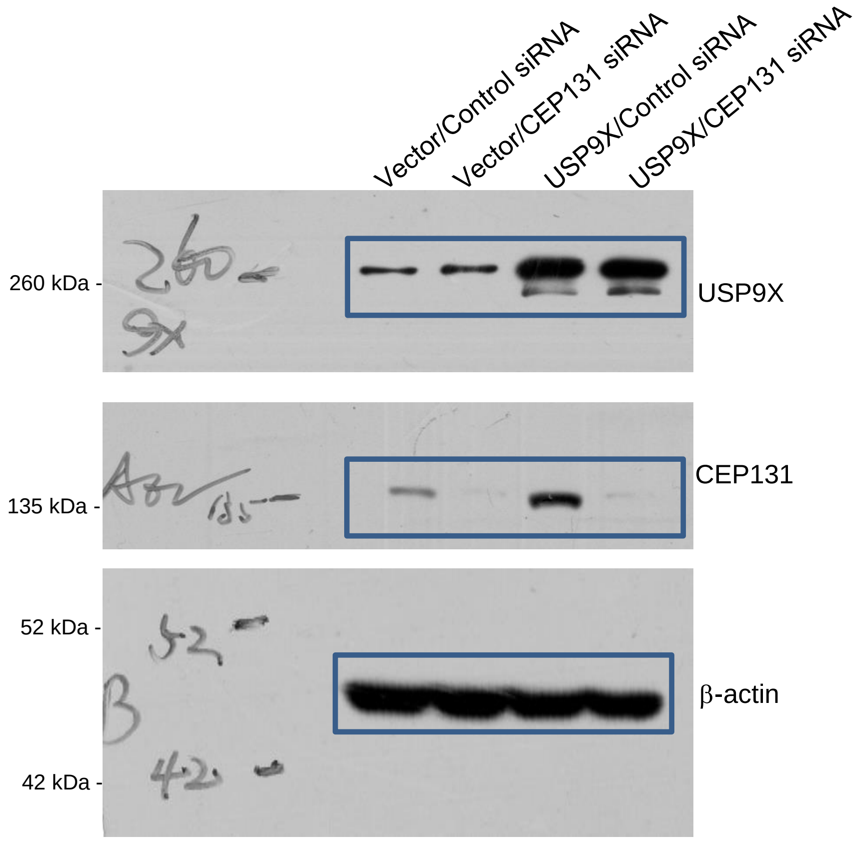

Uncropped blots related to Figure 5c

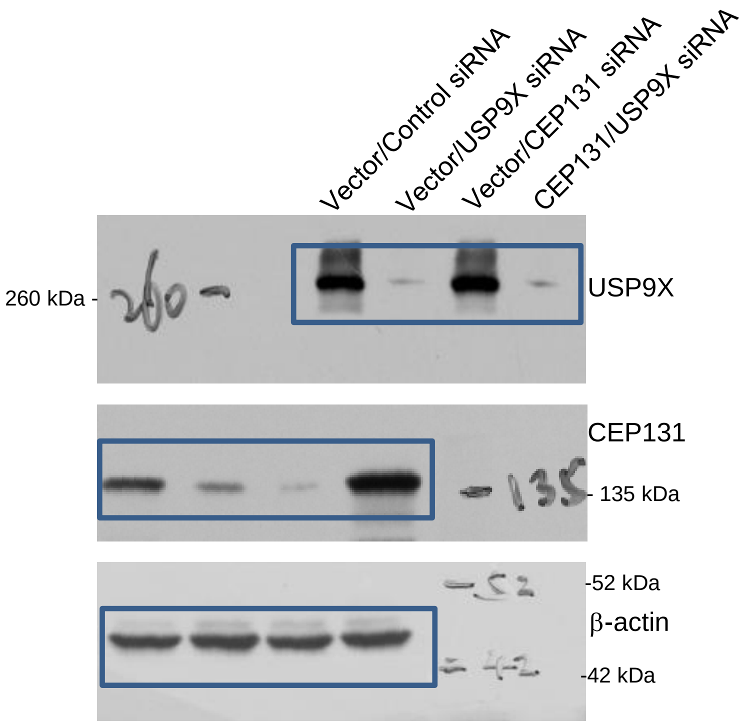

Uncropped blots related to Figure 5d

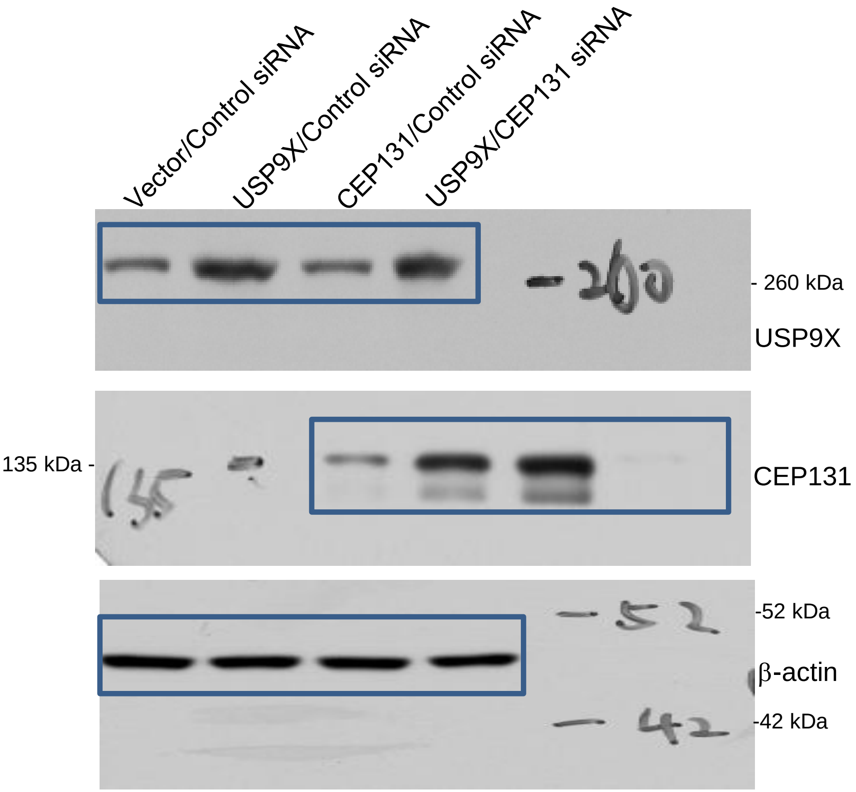

Uncropped blots related to Figure 6c

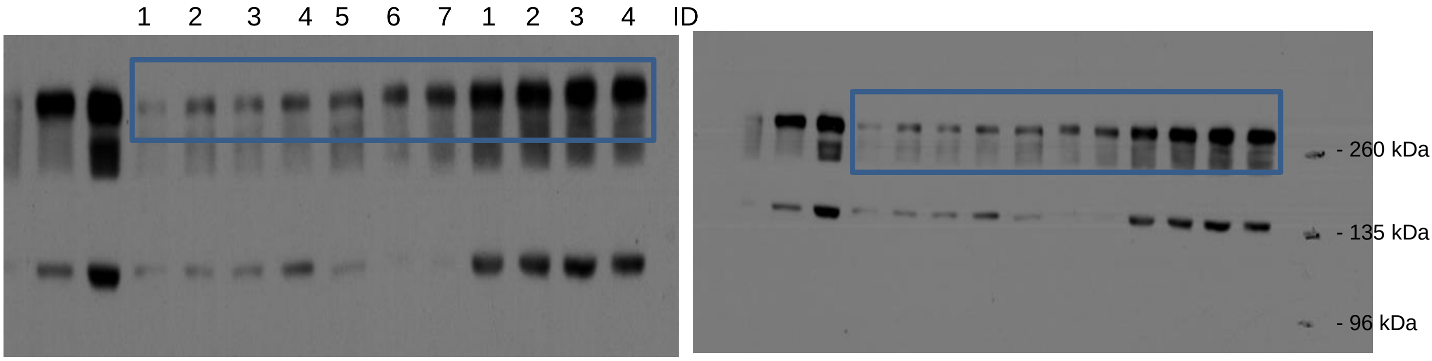

USP9X

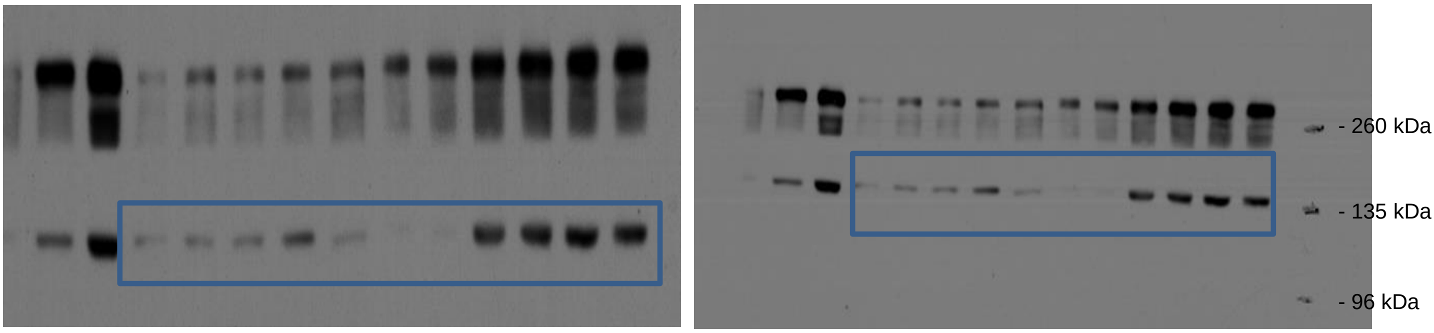

CEP131

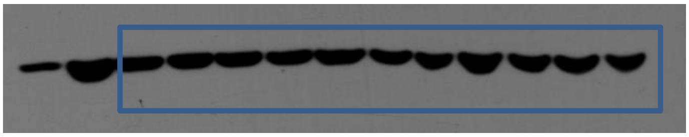

β-actin

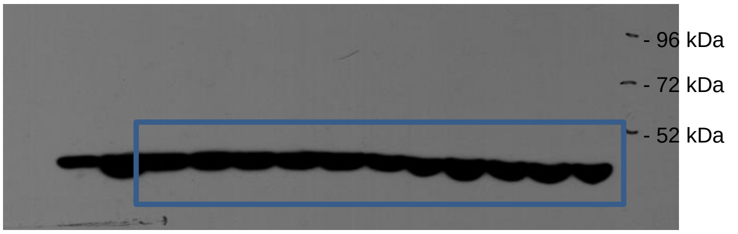

Uncropped blots related to Figure 6c

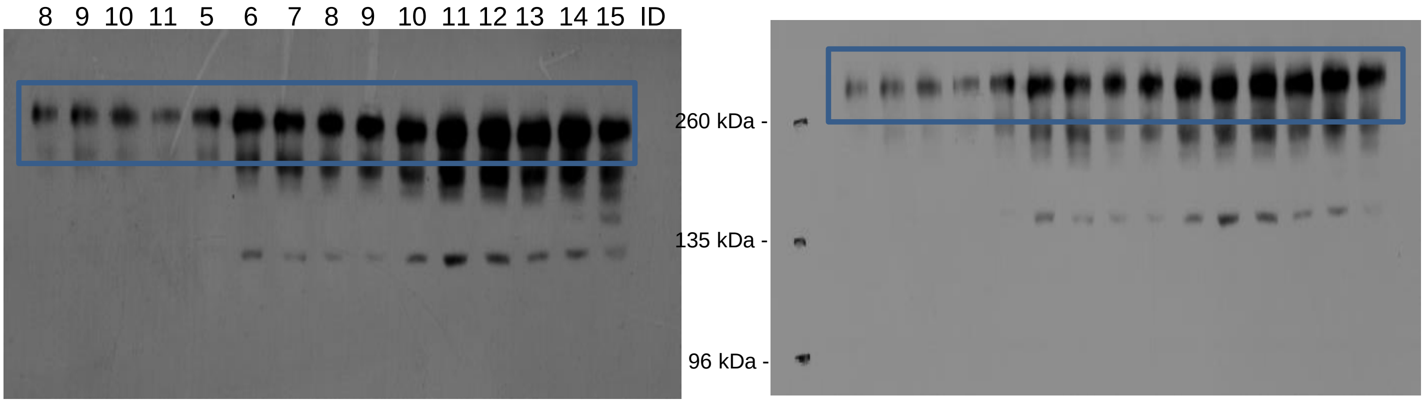

USP9X

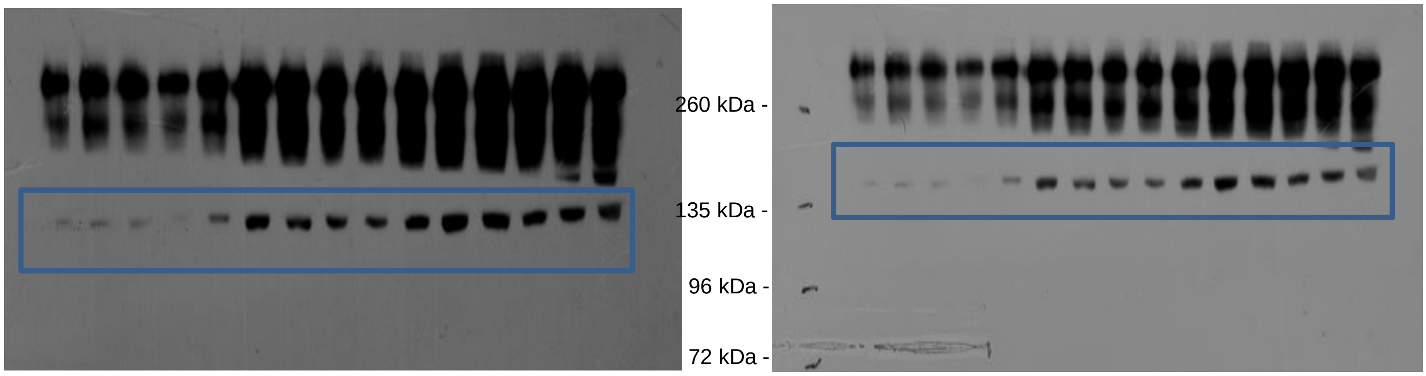

CEP131

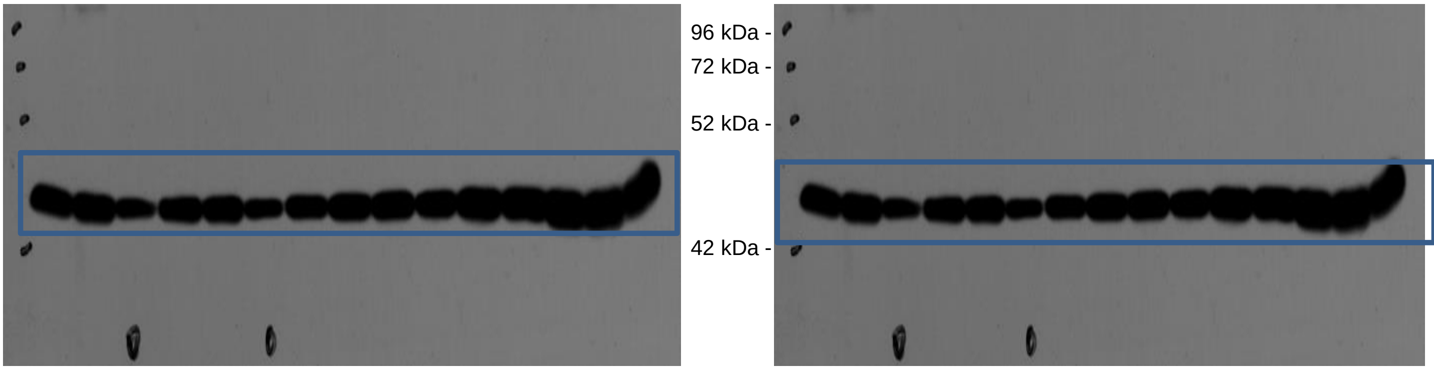

β-actin

Uncropped blots related to Figure 7b, upper panel

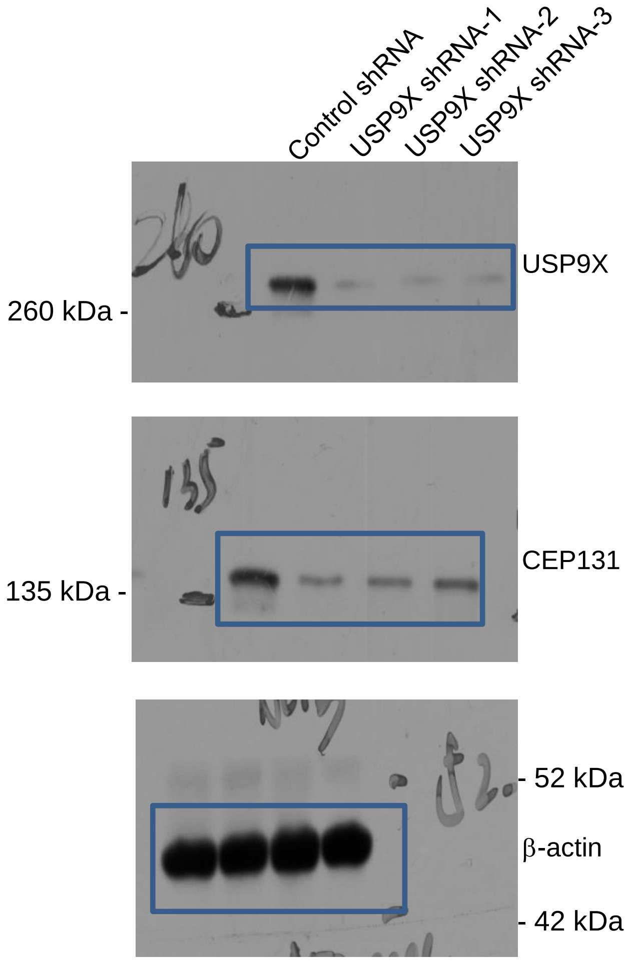

Uncropped blots related to Figure 7b, lower panel

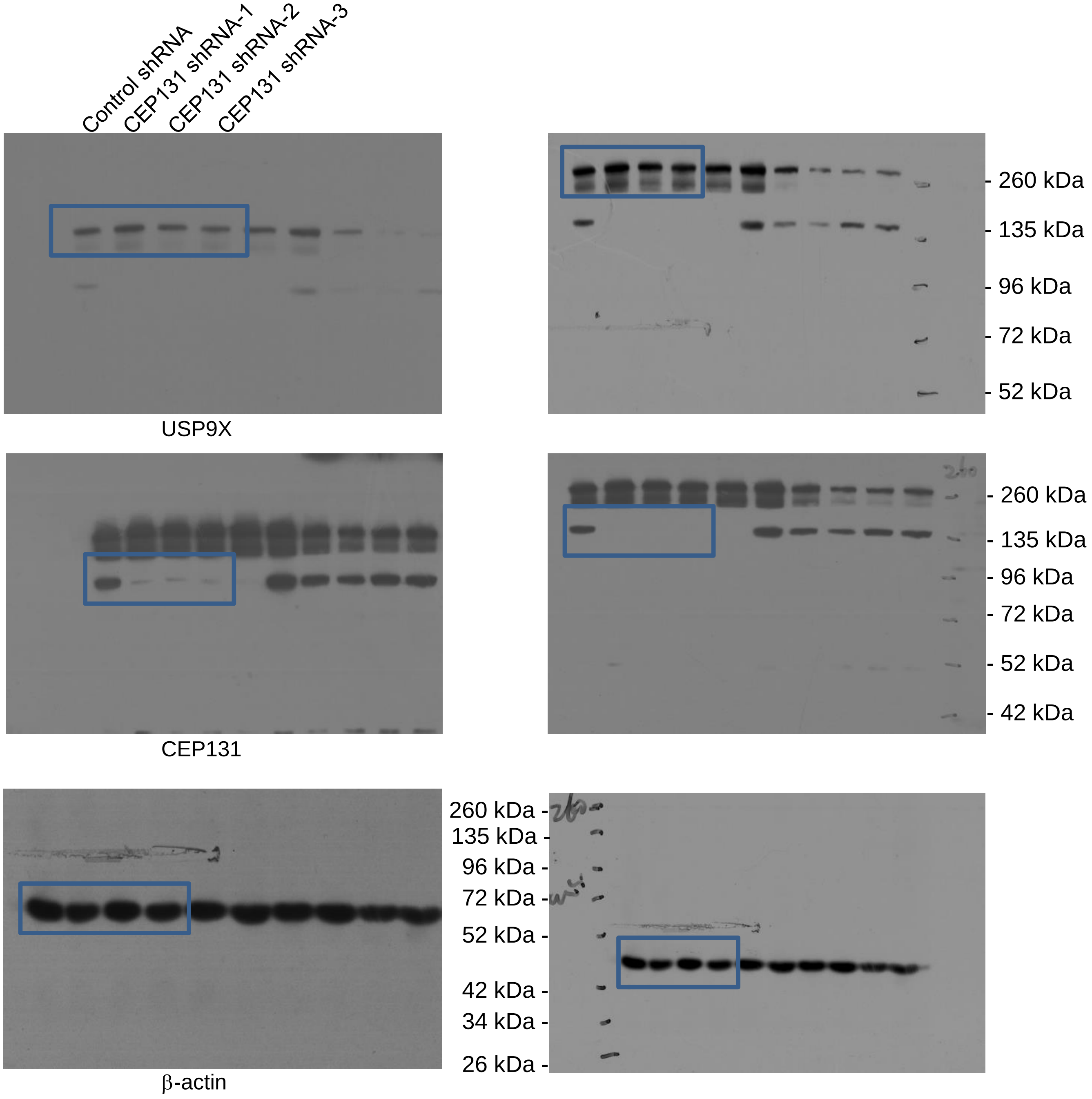

Uncropped blots related to Figure 7c

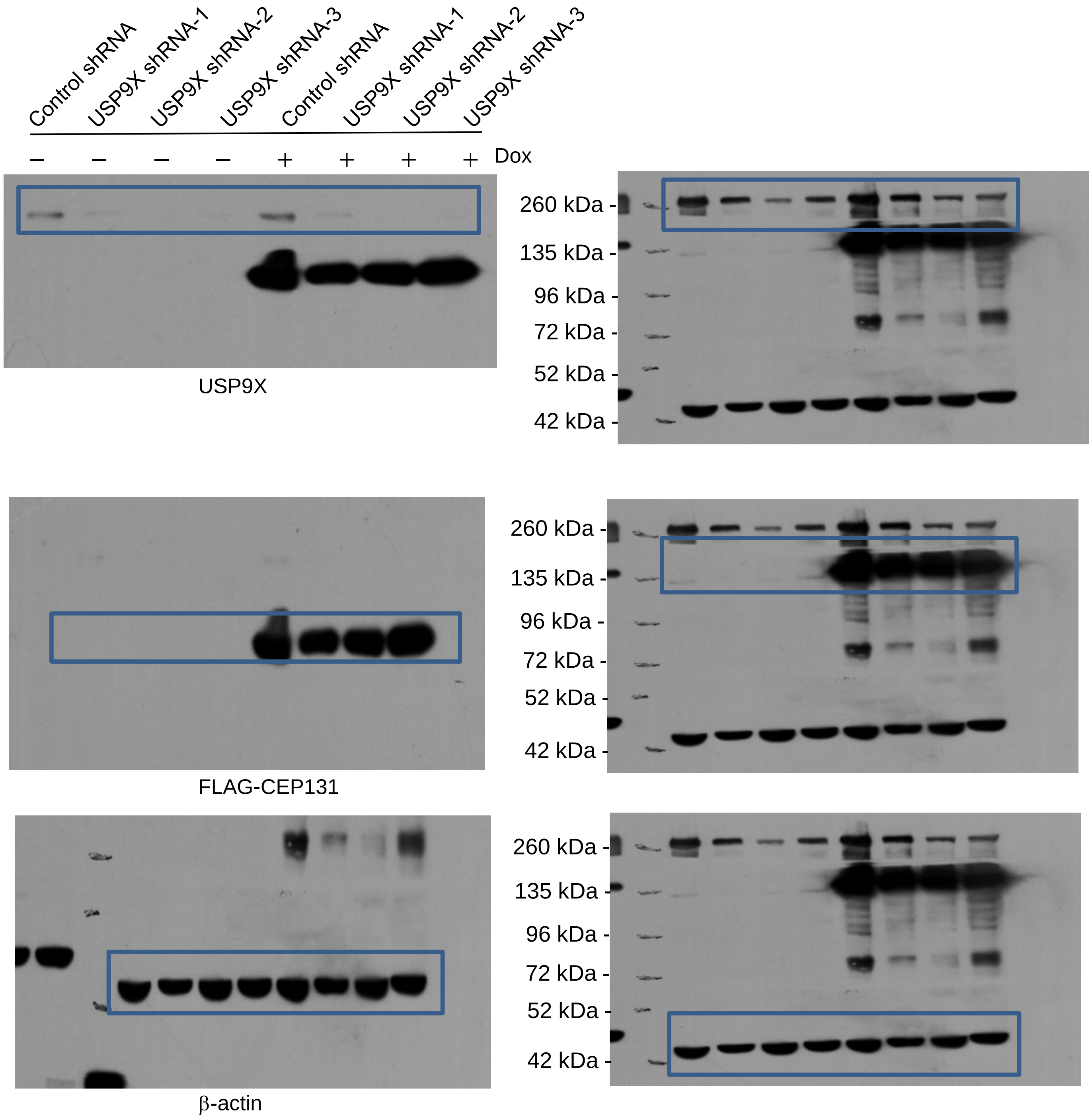

Uncropped blots related to Figure 7d

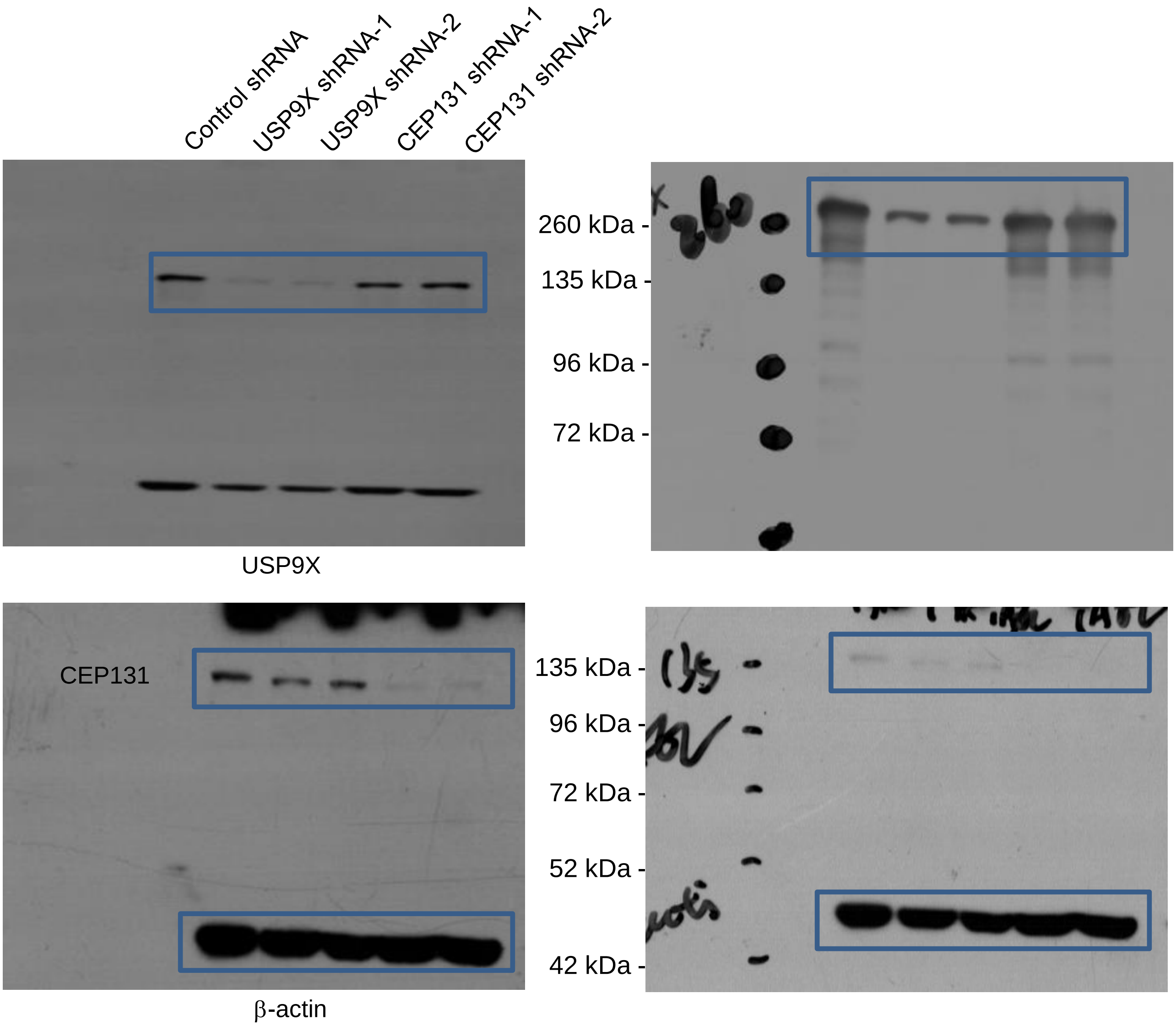

Uncropped blots related to Figure 7e

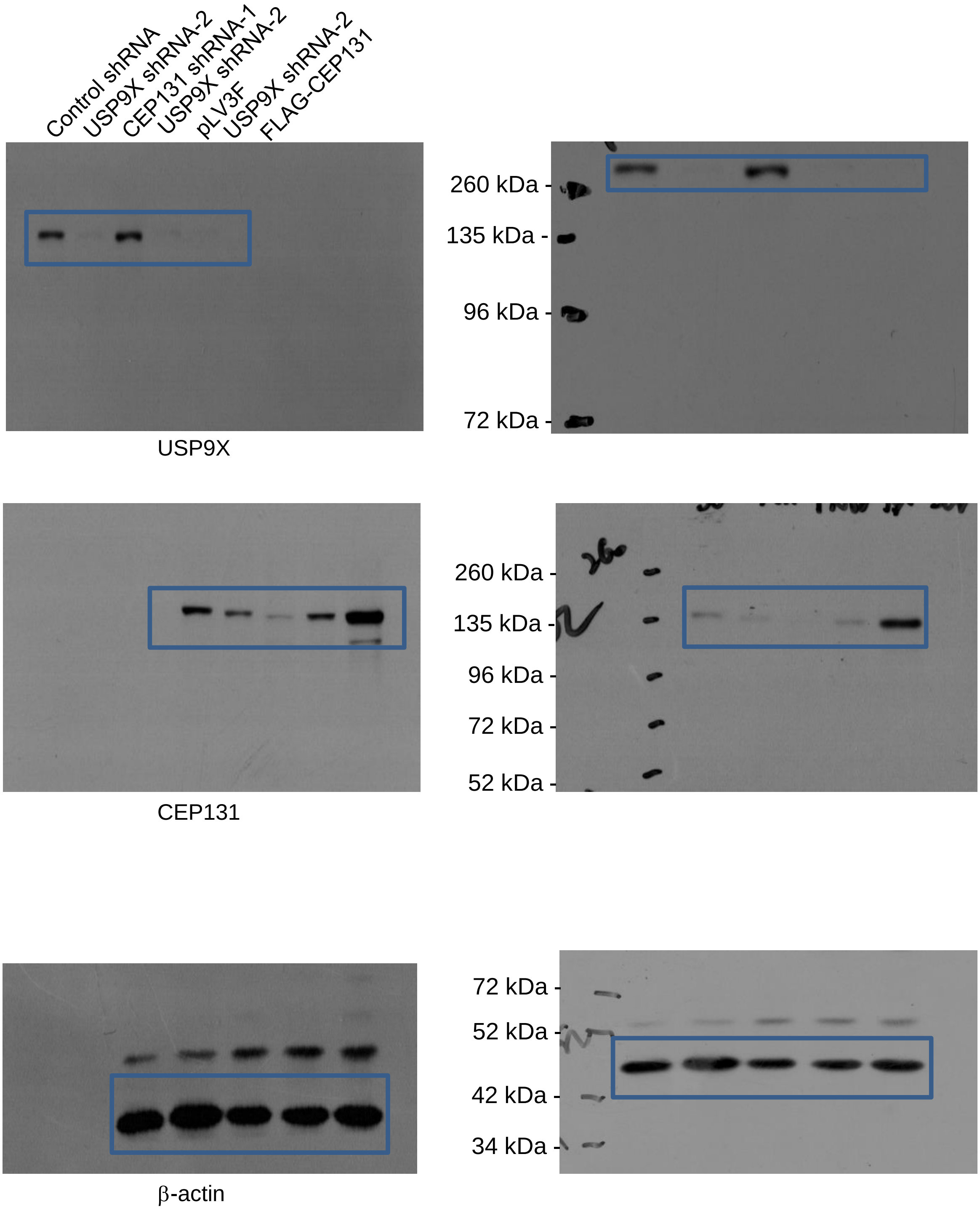

Supplementary Figure 11. Uncropped Scans for Figures 1-7. The relevant figures are indicated in the blot titles. In some blots, both lower and higher exposed ones with markers are provided. The cropped areas within the blue boxes are indicated.

Supplementary Table 1. Lentiviral shRNA sequences

shRNAs	Sequences
Luciferase	CCGGGATATGGGCTGAATACAAACTCGAGTTTGTATTCAGCCCATATCTTTTTG
USP9X-1	CCGGCGATTCTTCAAAGCTGTGAATCTCGAGATTCACAGCTTTGAAGAATCGTTTTTG
USP9X-2	CCGGGGTCGTTACAGCTAGTATTTACTCGAGTAAATACTAGCTGTAACGACCTTTTTG
USP9X-3	CCGGGAGAGTTTATTCACTGTCTTACTCGAGTAAGACAGTGAATAAACTCTCTTTTTG (3'UTR)
CEP131-1	CCGGGAGGAGGCTGAGAGGTTTATCCTCGAGGATAAACCTCTCAGCCTCCTCTTTTTG
CEP131-2	CCGGAGGCCCTCAAGGCCAACAATACTCGAGTATTGTTGGCCTTGAGGGCCTTTTTG
CEP131-3	CCGGCAGCACGAGCTGGAGATTAAACTCGAGTTTAATCTCCAGCTCGTGCTGTTTTTG

Note: Red color indicates the targeting sequence against the corresponding genes.

Supplementary Table 2. qRT-PCR primers

Genes	Sequences
PUM1	F: ATGAGCGTTGCATGTGTCTTG
	R: GTAGTCCACCATAGCGTCGTC
CEP131	F: TGATGCTCTTCGAGGGCAG
	R: GGAACTCCGGGCATTGGAT
USP9X	F: AAGTGAAGCATGTCAGCGATT
	R: GCCACACATAGCTCCACCA
PCM1	F: CGGAGTCGTCACCAGGAGT
	R: GCTGTTGCTCTACCTTGGGAAT
CEP290	F: AGATGCTCACCGAACAAGTAGA
	R: ATGAGTCTGTTGAGAAAGGGTTG
Pericentrin	F: TTGTGGTCCCAGCTTGATTCT
	R: CTCCAGTTCGGACTCATGCT
GAPDH	F: GAAGGTGAAGGTCGGAGTC
	R: GAAGATGGTGATGGGATTTC
